# Multi-variate model of T cell clonotype competition and homeostasis

**DOI:** 10.1038/s41598-023-46637-4

**Published:** 2023-12-11

**Authors:** Daniel Luque Duque, Jessica A. Gaevert, Paul G. Thomas, Martín López-García, Grant Lythe, Carmen Molina-París

**Affiliations:** 1 Department of Applied Mathematics, School of Mathematics, University of Leeds, Leeds, LS2 9JT UK; 2 Department of Immunology, St. Jude Children’s Research Hospital, Memphis, TN 38105 USA; 3 St. Jude Graduate School of Biomedical Sciences, Memphis, TN 38105 USA; 4 T-6, Theoretical Biology and Biophysics, Theoretical Division, Los Alamos National Laboratory, Los Alamos, NM 87545 USA

**Keywords:** Immunology, Applied mathematics, Population dynamics

## Abstract

Diversity of the naive T cell repertoire is maintained by competition for stimuli provided by self-peptides bound to major histocompatibility complexes (self-pMHCs). We extend an existing bi-variate competition model to a multi-variate model of the dynamics of multiple T cell clonotypes which share stimuli. In order to understand the late-time behaviour of the system, we analyse: *(i)* the dynamics until the extinction of the first clonotype, *(ii)* the time to the first extinction event, *(iii)* the probability of extinction of each clonotype, and *(iv)* the size of the surviving clonotypes when the first extinction event takes place. We also find the probability distribution of the number of cell divisions per clonotype before its extinction. The mean size of a new clonotype at quasi-steady state is an increasing function of the stimulus available to it, and a decreasing function of the fraction of stimuli it shares with other clonotypes. Thus, the probability of, and time to, extinction of a new clonotype entering the pool of T cell clonotypes is determined by the extent of competition for stimuli it experiences and by its initial number of cells.

## Introduction

An adult human has approximately $4\times 10^{11}$ T cells^1^, each of them expressing about $3\times 10^4$ identical T cell receptors (TCRs) on its surface^2^. These receptors recognise self-peptides bound to major histocompatibility complexes (MHCs), which as bound binary complexes are called self-pMHCs. The interaction between TCRs and self-pMHCs induces a T cell to synthesise proteins important for survival and homeostatic proliferation^3–6^. While the number of distinct TCRs in the naive T cell repertoire^7^ is at least $10^{7}$–$10^{8}$, the total number of T cells is such that most TCRs are present on many different T cells. These sub-populations of T cells sharing the same TCR are called T cell clonotypes. If we consider a single naive T cell clonotype with relatively little competition for self-pMHCs with other clonotypes, then its population dynamics can be modelled as a uni-variate birth and death process^8^. Thus, for these TCR clonotypes, self-pMHC stimulation promotes establishment in the periphery^9–12^. Competition implies that clonotypes are susceptible to extinction.

TCRs are inherently cross-reactive: one TCR can interact with many different pMHCs^13^. Individual TCRs have been estimated to recognize $10^6$ different pMHCs^14^, suggesting an overlap in the sets of self-pMHCs that stimulate different T cell clonotypes. Without this overlap, extinction of a clonotype would decrease the coverage of the TCR repertoire over the space of foreign peptides, which is known to be maintained even in the presence of such extinction events^15,16^.

A similar mathematical model can be used for two clonotypes which compete for self-pMHC survival stimuli. In this case, a bi-variate Markov competition process can be defined as in Ref.^17^. This bi-variate model can be used to show that extinction, for sufficiently late times, is certain for both clones, i.e., after a transient time one clonotype will become extinct and the remaining one will behave as described by the uni-variate model^8^. This is a closer representation of the competition for survival stimuli experienced by the naive T cell repertoire^11,12^. However, the highly oligoclonal nature of immune responses^18^ and the occurrence of similar TCRs^19^ serve as evidence that the self-pMHC recognition profile overlap will typically extend to more than two clonotypes.

Here, we propose a generalisation of the model presented in Ref.^17^ to characterise the competition of a number, $\eta$, of different T cell clonotypes ($\eta \ge 3$) with significant self-pMHC recognition overlap. Significant in this context means that the number of self-pMHCs shared by the clonotypes under consideration is so large that they cannot be modelled as single clonotypes^8^, nor as pair-wise competitors^17^. It is assumed that naive T cells of a given clonotype exit the thymus at roughly the same time^20^, and that after this time they are not generated again by the thymus, given the potential diversity of recombination^21,22^. Thus, the population dynamics of a naive T cell clonotype in the periphery depends on its homeostatic birth and death rates, and its extinction is possible^20^. Here, we will show that extinction of any clonotype is certain for sufficiently late times, and thus, some time after its thymic output into the periphery, there will be one fewer clonotype competing for stimuli. Mathematically this decrease in the number of competing clonotypes would continue until two remain, and finally until only one is left, taking us back to the models described in Refs.^8,17^, respectively.

Our main interest is in the perturbation of established clonotypes in the periphery, specifically by the introduction of a new one that competes with them. We study the dynamics of competition before the extinction of the first clonotype, and the population distribution after the first extinction event. This is relevant both if (1) the first extinction event corresponds to the clonotype that most recently arrived in the periphery, since it informs us on how its introduction modifies already homeostatically established clonotypes at both short (before extinction), and long (after extinction) timescales, or if (2) the first extinction event corresponds to an established peripheral clonotype, since it informs us on the probability a newly arrived clonotype has to become established.

In “Stochastic multi-variate model of naive T cell clonotype competition for self-pMHC stimuli” we introduce the competition model which describes the population of a number, $\eta$, of different naive T cell clonotypes, as well as the recognition tripartite network of self-pMHCs used to calculate their division (birth) rates. We also consider two special cases of clonotype competition in the periphery. “Quasi-stationary probability distribution” focuses on the quasi-stationary probability distribution (QSD), which we approximate making use of two additional processes: one in which extinction is not possible, and another where each clonotype has one immortal cell^23,24^. In “Study of clonal extinction” we prove that for sufficiently late (but finite) times, all clonotypes will become extinct. We also introduce the stochastic descriptors used to study the behaviour of the competition around these extinction events. Finally, in “Results” we use the QSD and the stochastic descriptors to analyse the perturbation exerted on two established clonotypes by a new clonotype entering the periphery. In this case we analyse four different competition scenarios for the three clonotypes. We also compare the behaviour of the competition process when the new clonotype is in both of the special cases discussed in “Stochastic multi-variate model of naive T cell clonotype competition for self-pMHC stimuli”. We end with a discussion in “Discussion”.

## Stochastic multi-variate model of naive T cell clonotype competition for self-pMHC stimuli

Let us consider two sets: the set, ${\mathcal {C}}$, of $\eta$ different clonotypes with a significant overlap in the self-pMHCs they recognise (see Fig. 1 of Ref.^25^), and ${\mathcal {Q}}$, the set of all self-pMHCs which can stimulate clonotypes in ${\mathcal {C}}$. We will describe the number of T cells belonging to each of the $\eta$ clonotypes at time *t* as a continuous time multi-variate Markov process, ${\mathcal {X}}=\left\{ (X_{1}(t),\ldots ,X_{\eta }(t)): t\ge 0\right\}$, over the state space ${\mathcal {S}}=\left\{ (n_{1},\ldots ,n_{\eta }): n_{i}\ge 0,\forall i\right\} ={\mathbb {N}}_{0}^{\eta }$, where $X_{i}(t)$ represents the number of cells of clonotype *i* at time *t* (for $1 \le i \le \eta$), and ${\textbf{X}}(t) = \left( X_{1}(t),\ldots ,X_{\eta }(t)\right)$ is the random vector describing the population of $\eta$ clonotypes being modelled at time *t*.

We assume that all cells of a particular clonotype exit the thymus at roughly the same time. However different clonotypes can exit the thymus at different times. Since we are interested in modelling the competition dynamics of all clonotypes in ${\mathcal {C}}$, we consider the initial time $t=0$ in our process ${\mathcal {X}}$ so that all clonotypes in ${\mathcal {C}}$ are already present in the periphery.

**Figure 1:**
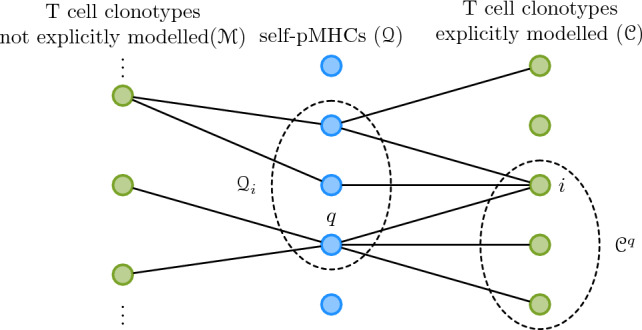
Tripartite network of TCR-self-pMHC recognition. Each blue circle represents a self-pMHC and each green circle a T cell clonotype. A clonotype is explicitly modelled if it is in ${\mathcal {C}}$, or part of the periphery but not explicitly modelled, if it is in ${\mathcal {M}}$. This implies ${\mathcal {C}}\cap {\mathcal {M}} = \emptyset$. An edge between a blue and a green circle represents the ability of that T cell clonotype to receive stimulus from the self-pMHC. For a given self-pMHC, $q\in {\mathcal {Q}}$, the set of clonotypes it can stimulate in ${\mathcal {C}}$ is ${\mathcal {C}}^{q}$, and for a given clonotype, $i\in {\mathcal {C}}$, the set of self-pMHCs that can stimulate it is ${\mathcal {Q}}_{i}$.

The division rate of cells belonging to a clonotype depends on the competition between clonotypes for shared self-pMHC stimuli^11,12,25,26^. To this end we consider a tripartite recognition network (see Fig. 1). In a recognition network each clonotype (green circle) is able to receive stimuli from a set of self-pMHCs (blue circles), and this ability is represented by an edge between the clonotype and the self-pMHC. We partition all peripheral naive T cell clonotypes as follows: clonotypes in the periphery are in ${\mathcal {C}}$, if they are explicitly modelled, or in ${\mathcal {M}}$, if they are not explicitly modelled. We note that this definition implies ${\mathcal {C}}\cap {\mathcal {M}} = \emptyset$. Each clonotype $i\in {\mathcal {C}}$ has an associated set of self-pMHCs that stimulate it, denoted by ${\mathcal {Q}}_{i}$ (see Fig. 1).

We assume all self-pMHCs provide the same (constant) rate of homeostatic proliferation stimulus, and denote it by $\gamma$^8,17^. Then, we can write the total homeostatic proliferation stimulus each naive T cell of clonotype *i* receives if the system of $\eta$ clonotypes is in state ${\textbf{n}}=(n_{1},\ldots ,n_{\eta })\in {\mathcal {S}}$ as follows

1 $$\begin{aligned} \Lambda ^{(i)}({\textbf{n}})=\sum _{q\in {\mathcal {Q}}_{i}}\frac{\gamma }{n_{q}}, \end{aligned}$$

where $n_{q}$ is the total number of naive T cells in the periphery (${\mathcal {C}}\cup {\mathcal {M}}$) that are stimulated by self-pMHC *q* (see Fig. 1). With this stimulus rate we can now define the birth rate of clonotype *i* in state ${\textbf{n}}\in {\mathcal {S}}$, as the transition rate from state ${\textbf{n}}$ to state ${\textbf{n}}^{(+i)}{:}{=}(n_{1},\ldots ,n_{i-1},n_{i}+1,n_{i+1},\ldots ,n_{\eta })$, which is given by

2 $$\begin{aligned} \lambda _{{\textbf{n}}}^{(i)}=n_{i}\Lambda ^{(i)}({\textbf{n}}). \end{aligned}$$

Similarly, the transition rate from state ${\textbf{n}}\in {\mathcal {S}}$ to state ${\textbf{n}}^{(-i)}{:}{=}(n_{i},\ldots ,n_{i-1},n_{i}-1,n_{i+1},\ldots ,n_{\eta })$ is the death rate of clonotype *i*, and it is given by

3 $$\begin{aligned} \mu _{{\textbf{n}}}^{(i)}=\mu _{i}n_{i}. \end{aligned}$$

If $n_{i}=0$ for any clonotype *i*, with $1 \le i \le \eta$, its birth and death rates will both be zero, in agreement with our assumption on thymic production. Thus, we observe that the set of states with at least one entry equal to zero, ${\mathcal {A}}=\left\{ (n_{1},\ldots ,n_{\eta })\in {\mathcal {S}}: n_{i}=0\text { for any }i\right\}$, with $1 \le i \le \eta$, is an absorbing set, and the state $(0,\ldots ,0)$ is an absorbing state representing the extinction of all $\eta$ clonotypes^27,28^.

Let us consider two states, ${\textbf{n}}=(n_{1},\ldots ,n_{\eta })$ and ${\textbf{m}}=(m_{1},\ldots ,m_{\eta })$ in ${\mathcal {S}}$. The transition probability from ${\textbf{n}}$ to ${\textbf{m}}$ in a small time interval, $\Delta t$, is defined as

$$\begin{aligned} p_{\textbf{nm}}(\Delta t)={\mathbb {P}}\left( {\textbf{X}}(t+\Delta t)={\textbf{m}} \bigg | \ {\textbf{X}}(t)={\textbf{n}}\right) , \end{aligned}$$

and in the limit $\Delta t\rightarrow 0^+$, this transition probability satisfies

$$\begin{aligned} p_{\textbf{nm}}(\Delta t)= {\left\{ \begin{array}{ll} \lambda _{{\textbf{n}}}^{(i)}\Delta t+o(\Delta t), &{}\quad \text {if} \; {\textbf{m}}={\textbf{n}}^{(+i)}, \\ \mu _{{\textbf{n}}}^{(i)}\Delta t+o(\Delta t), &{}\quad \text {if} \; {\textbf{m}}={\textbf{n}}^{(-i)}, \\ 1 -\sum _{i=1}^{\eta }(\lambda _{{\textbf{n}}}^{(i)}+\mu _{{\textbf{n}}}^{(i)})\Delta t +o(\Delta t), &{}\quad \text {if} \; {\textbf{m}}={\textbf{n}}, \\ o(\Delta t), &{}\quad \text {otherwise}. \end{array}\right. } \end{aligned}$$

The clonotypes in ${\mathcal {C}}$ are explicitly modelled by the process ${\mathcal {X}}$, yet there are other clonotypes in the naive T cell repertoire which can also receive stimuli from self-pMHCs in ${\mathcal {Q}}$, but which do not overlap significantly with the clonotypes in ${\mathcal {C}}$. These clonotypes are contributing to the competition for stimuli as a “sink”, in the sense that they are taking a portion of the stimuli, but their population dynamics is not explicitly modelled. We denote the set of these clonotypes by ${\mathcal {M}}$ and define its cardinality to be $\vert {\mathcal {M}}\vert =M$. We define ${\mathcal {C}}^{q}$ as the set of clonotypes in ${\mathcal {C}}$ which can receive stimuli from self-pMHC $q\in {\mathcal {Q}}$, and separate $n_{q}$ into the number of cells in ${\mathcal {C}}^{q}$, which receive stimuli from self-pMHC *q*, and the number of cells in ${\mathcal {M}}$, which receive stimuli from self-pMHC *q*. Since we do not explicitly model the cells in ${\mathcal {M}}$, we will assume these populations are homeostatically established^8^, and thus, have a constant size (see Eq. (A.9) in the Supplementary Material).

### Approximation of the transition rates

In what follows we illustrate the definition of the transition rates in the case $\eta =3$; that is, the case where only three clonotypes are explicitly modelled. For the general formulation of these rates, details have been provided in Appendix A (see Supplementary Material). We focus on this specific case for two main reasons. First, when considering the generalisation of a two-dimensional competition model, a three-dimensional model is the simplest case to be studied. Furthermore, as shown in Appendices E, F, and G in the Supplementary Material, after a finite transient time a clonotype will become extinct, and the competition process can be modelled using a process with dimension one fewer than the original. Second, the computational cost of calculating quantities of interest for a model with more than three clonotypes is presently too high, making parameter exploration, and analysis of such scenarios intractable.

We first write Eq. (1) making use of the following definition. Let $n_{iq}$, for $i=1,2,3$ and $q\in {\mathcal {Q}}_{i}$, be the number of cells not of clonotype *i* that can receive stimulus from self-pMHC *q*; that is, $n_{iq}=n_{q}-n_{i}$ for $i=1,2,3$, so that Eq. (1) becomes

4 $$\begin{aligned} \Lambda ^{(i)}({\textbf{n}})=\sum _{q\in {\mathcal {Q}}_{i}}\frac{\gamma }{n_{i}+n_{iq}}, \quad \text {for } i=1,2,3. \end{aligned}$$

By writing the per-cell birth rate in this manner it is easy to see that it depends not only on clonotype *i*, but on all other clonotypes which compete for stimuli from self-pMHCs in ${\mathcal {Q}}_{i}$. In addition, we can subdivide the set of self-pMHCs that can stimulate clonotype *i* into sets ${\mathcal {Q}}_{ij}^{k}$, where *j* denotes the number of other clonotypes that the stimulus is shared with (including the case $j=0$, where the stimulus is not shared with other clonotypes), and *k* indexes the different possible choices of *j* clonotypes (see Eq. (A.2) and Eq. (A.3) in the Supplementary Material). Then, using these subsets we can write Eq. (4) for clonotype 1 as follows

5 $$\begin{aligned} \begin{aligned} \Lambda ^{(1)}({\textbf{n}})&= \sum _{q\in {\mathcal {Q}}_{1,0}^{1}}\frac{\gamma }{n_{1}+n_{1q}} + \sum _{q\in {\mathcal {Q}}_{1,1}^{1}}\frac{\gamma }{n_{1}+n_{2}+n_{12q}} \\&\quad + \sum _{q\in {\mathcal {Q}}_{1,1}^{2}}\frac{\gamma }{n_{1}+n_{3}+n_{13q}} + \sum _{q\in {\mathcal {Q}}_{1,2}^{1}}\frac{\gamma }{n_{1}+n_{2}+n_{3}+n_{123q}}. \end{aligned} \end{aligned}$$

We now take into account the set of clonotypes not explicitly modelled, ${\mathcal {M}}$. Thus, we can further partition the stimulus shared by clonotype 1 with clonotypes 2 and 3 by the number of clonotypes in ${\mathcal {M}}$ with which the stimulus is being shared (see Eq. (A.6) in the Supplementary Material). Using this partition of the stimulus we can then consider a mean field approximation (see Eq. (A.8) in the Supplementary Material) and Ref.^8^), which can be simplified by making use of the Poisson approximation for binomial distributions (see Eq. (A.11) and Eq. (A.12) in the Supplementary Material). By doing so we introduce two new parameters: the mean niche overlap $\nu _{ij}^{k}$, which represents the expected number of clonotypes in ${\mathcal {M}}$ that receive stimulus from self-pMHCs in ${\mathcal {Q}}_{ij}^{k}$, and $\langle n\rangle$, the average number of cells for each clonotype in ${\mathcal {M}}$. Then, the per-cell birth rate of clonotype 1 can be approximated as

6 $$\begin{aligned} \begin{aligned} \Lambda ^{(1)}({\textbf{n}})&= \varphi _{1}\cdot p_{1,0}^{1}\cdot e^{-\nu _{1,0}^{1}}\sum _{r=0}^{M}\frac{\left( \nu _{1,0}^{1}\right) ^{r}}{r!}\frac{1}{n_{1}+r\langle n\rangle } \\&\quad + \varphi _{1}\cdot p_{1,1}^{1}\cdot e^{-\nu _{1,1}^{1}}\sum _{r=0}^{M}\frac{\left( \nu _{1,1}^{1}\right) ^{r}}{r!}\frac{1}{n_{1}+n_{2}+r\langle n\rangle } \\&\quad + \varphi _{1}\cdot p_{1,1}^{2}\cdot e^{-\nu _{1,1}^{2}}\sum _{r=0}^{M}\frac{\left( \nu _{1,1}^{2}\right) ^{r}}{r!}\frac{1}{n_{1}+n_{3}+r\langle n\rangle } \\&\quad + \varphi _{1}\cdot p_{1,2}^{1}\cdot e^{-\nu _{1,2}^{1}}\sum _{r=0}^{M}\frac{\left( \nu _{1,2}^{1}\right) ^{r}}{r!}\frac{1}{n_{1}+n_{2}+n_{3}+r\langle n\rangle }, \end{aligned} \end{aligned}$$

where $\varphi _{1}$ is the total stimulus available to clonotype 1, and $p_{1,j}^{k}$ is the probability of a self-pMHC in ${\mathcal {Q}}_{1}$ being in ${\mathcal {Q}}_{1,j}^{k}$; that is, $\varphi _{1}=\gamma \vert {\mathcal {Q}}_{1}\vert$, and $p_{1,j}^{k}=\vert {\mathcal {Q}}_{1,j}^{k}\vert /\vert {\mathcal {Q}}_{1}\vert$. We note that given these definitions of $\varphi _{i}$ and $p_{ij}^{k}$, we have the following relations (see Eq. (A.15) in the Supplementary Material):

7 $$\begin{aligned} \varphi _{1}p_{1,2}^{1}= & {} \varphi _{2}p_{2,2}^{1} = \varphi _{3}p_{3,2}^{1}, \nonumber \\ \varphi _{1}p_{1,1}^{1}= & {} \varphi _{2}p_{2,1}^{1}, \nonumber \\ \varphi _{1}p_{1,1}^{2}= & {} \varphi _{3}p_{3,1}^{1}, \nonumber \\ \varphi _{2}p_{2,1}^{2}= & {} \varphi _{3}p_{3,1}^{2}. \end{aligned}$$

Finally, the per-cell birth rate shown in Eq. (6) can be simplified for two limiting cases. The first one when $\nu _{ij}^{k}\ll 1$ for all $\nu _{ij}^{k}$, called the “hard niche” limit, which is characterised by weak competition with clonotypes not explicitly modelled. In this case the birth rate for clonotype 1 simplifies to

8 $$\begin{aligned} \lambda ^{(1)}_{{\textbf{n}}} = \varphi _{1}n_{1}\left( \frac{p_{1,0}^{1}}{n_{1}}+\frac{p_{1,1}^{1}}{n_{1}+n_{2}}+\frac{p_{1,1}^{2}}{n_{1}+n_{3}}+\frac{p_{1,2}^{1}}{n_{1}+n_{2}+n_{3}}\right) . \end{aligned}$$

The second case, where $\nu _{ij}^{k}\gg 1$ for all $\nu _{ij}^{k}$, called the “soft niche”, in which there is significant competition with clonotypes not explicitly modelled. In this case the birth rate of clonotype 1 is approximated by

9 $$\begin{aligned} \begin{aligned} \lambda ^{(1)}_{{\textbf{n}}}&\approx \varphi _{1}n_{1}\left( \frac{p_{1,0}^{1}}{n_{1}+\nu _{1,0}^{1}\langle n\rangle }+\frac{p_{1,1}^{1}}{n_{1}+n_{2}+\nu _{1,1}^{1}\langle n\rangle }\right. \\&\quad \left. +\frac{p_{1,1}^{2}}{n_{1}+n_{3}+\nu _{1,1,}^{2}\langle n\rangle }+\frac{p_{1,2}^{1}}{n_{1}+n_{2}+n_{3}+\nu _{1,2}^{1}\langle n\rangle }\right) . \end{aligned} \end{aligned}$$

## Quasi-stationary probability distribution

We now want to study the behaviour of $\eta$ competing clonotypes before the first extinction event occurs. In order to do so, we introduce the quasi-stationary probability distribution (QSD), which describes the late time behaviour of the process conditioned on non-extinction^23,29,30^. We introduce $p_{{\textbf{n}}}(t)$, the probability that at time *t* the competition process ${\mathcal {X}}$ is in state ${\textbf{n}}$, given that it started in state ${\textbf{n}}_{0}$, i.e.,

10 $$\begin{aligned} p_{{\textbf{n}}}(t)={\mathbb {P}}\left( {\textbf{X}}(t)={\textbf{n}} \bigg | \ {\textbf{X}}(0)={\textbf{n}}_{0}\right) . \end{aligned}$$

Let us consider the absorbing set ${\mathcal {A}}=\left\{ (n_{1},\ldots ,n_{\eta }): n_{i}=0\text { for any }i\right\}$ and denote by $p_{{\overline{{\mathcal {A}}}}}(t)$ the probability that at time *t* the process is not in ${\mathcal {A}}$. Now, we define the probability that the process is in state ${\textbf{n}}\in {\mathcal {S}}\setminus {\mathcal {A}}$ at time *t* given that absorption into ${\mathcal {A}}$ has not occurred yet, as follows

11 $$\begin{aligned} q_{{\textbf{n}}}(t)={\mathbb {P}}\left( {\textbf{X}}(t) = {\textbf{n}}\ \bigg |\ {\textbf{X}}(t) \notin {\mathcal {A}}\right) = \frac{p_{{\textbf{n}}}(t)}{p_{{\overline{{\mathcal {A}}}}}(t)}. \end{aligned}$$

Finding an analytical solution for the probabilities defined in Eq. (11) is in general not possible (see Appendix B in the Supplementary Material), and thus, the QSD will be numerically approximated. We discuss two useful approximations in the following section, which were proposed in Refs.^23,24^.

### Approximation of the QSD: two auxiliary processes

We approximate the QSD making use of two auxiliary competition processes^24^. In the first approximation we will consider the multi-variate Markov process ${\mathcal {X}}^{(1)}=\left\{ \left( X_{1}^{(1)}(t),\ldots ,X_{\eta }^{(1)}(t)\right) : t\ge 0\right\}$, where $X_{i}^{(1)}(t)$ is the number of cells of clonotype *i* at time *t*. The birth rate of clonotype *i* in state ${\textbf{n}}=(n_{1},n_{2},\ldots ,n_{\eta })$, $\lambda ^{1,(i)}_{{\textbf{n}}}$, is given by Eq. (A.14) in the Supplementary Material, and its death rate by

$$\begin{aligned} \mu _{{\textbf{n}}}^{1,(i)}= {\left\{ \begin{array}{ll} \mu _{i}n_{i}, &{}\quad \text { if } n_{i}>1, \\ 0, &{}\quad \text { if } n_{i}=1. \end{array}\right. } \end{aligned}$$

This process, so defined, makes the extinction of clonotypes impossible. Thus, the state space of ${\mathcal {X}}^{(1)}$ is the set of states where no extinction has occurred

12 $$\begin{aligned} {\mathcal {A}}^{0}= {\mathcal {S}}\setminus {\mathcal {A}}= \left\{ {\textbf{n}}\in {\mathcal {S}}: n_{i}>0\text { for all } 1 \le i \le \eta \right\} . \end{aligned}$$

The second process we will consider is ${\mathcal {X}}^{(2)}=\left\{ \left( X_{1}^{(2)}(t),\ldots ,X_{\eta }^{(2)}(t)\right) : t\ge 0\right\}$, where the birth rates, $\lambda ^{2,(i)}_{{\textbf{n}}}$, are the same as those for ${\mathcal {X}}$ and ${\mathcal {X}}^{(1)}$, and we consider an immortal cell present in each clonotype; that is, the death rates are given by

$$\begin{aligned} \mu _{{\textbf{n}}}^{2,(i)}=\mu _{i}(n_{i}-1), \end{aligned}$$

and the state space of ${\mathcal {X}}^{(2)}$ is also ${\mathcal {A}}^{0}$.

To approximate the QSD for a multi-variate competition system, we will calculate the stationary probability distribution of the two auxiliary processes defined above^23,24^. To this end we first separate ${\mathcal {A}}^{0}$ into subsets, which we will call levels, as follows

$$\begin{aligned} L^{0}(k)=\left\{ (n_{1},\ldots ,n_{\eta }):\sum _{i=1}^{\eta }n_{i}=k \text { and } n_{i}>0 \text { for all } i\right\} , \end{aligned}$$

for $k=\eta ,\eta +1,\eta +2,\ldots$. Then, these levels can be ordered as follows

$$\begin{aligned} L^{0}(\eta )\prec L^{0}(\eta +1)\prec L^{0}(\eta +2)\prec L^{0}(\eta +3)\prec \cdots , \end{aligned}$$

and the states in each level can be ordered using the colexico-graphical order, sometimes called reverse lexico-graphical order^31^ (see Appendix C in the Supplementary Material). Note that we start at $L^{0}(\eta )$, since any state with fewer than $\eta$ cells in total does not belong to ${\mathcal {A}}^{0}$ by definition. Using a combinatorial argument^32^, we find that

13 $$\begin{aligned} L^{0}_{k} {:}{=}\vert L^{0}(k)\vert = {k-1\atopwithdelims ()\eta -1}. \end{aligned}$$

We now introduce the plane $\sum _{i=1}^{\eta }n_{i}=N$ as a reflecting boundary on our state space. This means that we only consider states which have at most *N* cells in total. In practice, this truncation value can be chosen so that the probability of exceeding a total number of cells, *N*, in the population is negligible (see “Study of clonal extinction”). Then, the number of states in ${\mathcal {A}}^{0}$ is

$$\begin{aligned} \vert {\mathcal {A}}^{0}\vert =\sum \limits _{i=\eta }^{N}L^{0}_{i}=\sum \limits _{i=\eta }^{N}{i-1\atopwithdelims ()\eta -1}={N\atopwithdelims ()\eta }. \end{aligned}$$

Finally, the stationary probability distributions of ${\mathcal {X}}^{(1)}$ and ${\mathcal {X}}^{(2)}$ can be computed with a linear level-reduction algorithm^33^ (see Appendix D in the Supplementary Material), an outline of which is given in Algorithm D.1 in the Supplementary Material.

## Study of clonal extinction

We next studied the behaviour of the competition process ${\mathcal {X}}$ and its extinction events with the use of stochastic descriptors and first step arguments. We first show that our system will reach the absorbing state with probability 1 (see Appendix E in the Supplementary Material); that is, all clonotypes are guaranteed to become extinct for late enough times. Then, we show that total extinction is not only guaranteed, but it occurs in finite time (see Appendix F in the Supplementary Material). However, it is important to note that this finite time could be on a timescale much longer than those considered in biology, making the populations effectively immortal on a biological timescale.

Since the mean time to the extinction of all clonotypes is finite, the mean time to the first extinction event is also finite. We found an expression for the mean time to the first extinction event (see Appendix G in the Supplementary Material), to understand the timescales at which a model with $\eta$ clonotypes can be simplified to a model with $\eta -1$ clonotypes. Following this, we studied the distribution of clonal sizes at the time of the first extinction event to study changes in the population of surviving clonotypes (see Appendix H in the Supplementary Material). In calculating this probability distribution, we obtain two important descriptors of the model. First, the probability distribution of clonal sizes at the time of the first extinction event ${\textbf{U}}$, which we can separate into the following probabilities

14 $$\begin{aligned} {\textbf{U}}^{i}_{{\textbf{n}}, {\textbf{m}}}={\mathbb {P}}\left(\begin{array}{c} {\text{ the process is at state }} {\textbf{m}} {\text{ at the}}\\ {\text{time of the first extinction event}}\end{array} \bigg | {\textbf{X}}(0)={\textbf{n}}, \begin{array}{c}{\text{ and clonotype i}}\\ {\text{became extinct}}\end{array}\right) , \end{aligned}$$

and second, the probabilities of each clonotype being the first to become extinct given an initial state ${\textbf{n}}$

15 $$\begin{aligned} {\mathcal {U}}_{{\textbf{n}}}^{i}={\mathbb {P}}\left(\begin{array}{c} {\text{ clonotype i is the first }}\\ {\text{ to become extinct}}\end{array} \bigg | {\textbf{X}}(0)={\textbf{n}} \right) . \end{aligned}$$

Finally, we studied the distribution of divisions before a clonotype becomes extinct given an initial state ${\textbf{n}}$ (see Appendix J in the Supplementary Material); that is, we considered

16 $$\begin{aligned} {\mathcal {D}}_{i,d}({\textbf{n}}) = {\mathbb {P}}\left( \begin{array}{c}{\text { cells of clone i divided d}} \\ {\text{times before clonal extinction}} \end{array}\bigg | {\textbf{X}}(0)={\textbf{n}}\right) , \end{aligned}$$

which can be used as a measure of proliferation of a T cell clonal family before its extinction.

## Results

We now focus our study to the dynamics of two established clonotypes, which compete for self-pMHC stimuli with a third, new, clonotype that has just exited the thymus. Thus, the two clonotypes will be in a state defined by the mean of their two-dimensional QSD (and in the absence of the newly arrived clonotype). In this case, there are only two $p_{ij}^{k}$ probabilities for each clonotype. The probability $p_{i,0}$ that a self-pMHC is recognised only by clonotype *i*, and the probability $p_{i,1}$ that a self-pMHC is recognised by both clonotypes. Thus, by Eq. (A.15)  in the Supplementary Material, we only need to choose a value for one of the probabilities to determine the rest.

**Figure 2:**
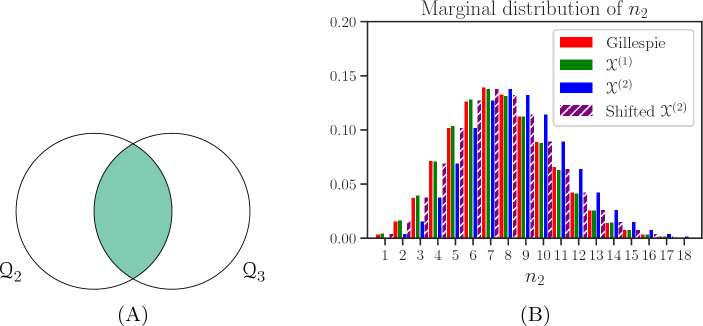
(**A**) Competition scenario considered for the two established clonotypes, where they compete for 1/3 of their stimulus. The shaded region represents the subset of self-pMHCs considered to be in the soft niche, with a mean niche overlap value $\nu _{2,1}=\nu _{3,1}=1$. (**B**) Marginal distribution of the QSD for clonotype 2, approximated using the processes ${\mathcal {X}}^{(1)}$ and ${\mathcal {X}}^{(2)}$ defined in “Approximation of the QSD: two auxiliary processes”.

We will denote the new clonotype as clonotype 1, and the established ones as clonotype 2 and clonotype 3, respectively. We consider all three clonotypes to have the same total homeostatic stimulus; that is, we assume $\varphi _{1}=\varphi _{2}=\varphi _{3}=10\text { cells}\cdot \text {year}^{-1}$^20^. Before the arrival of clonotype 1 we hypothesise ${\mathcal {Q}}_{2}\cap {\mathcal {Q}}_{3}$ to be in the *soft* niche, and the remaining self-pMHCs in the *hard* niche. We remind the reader that “hard niche” implies little competition with clonotypes not explicitly modelled, and “soft niche” that self-pMHCs are significantly competed for with clonotypes not explicitly modelled. Figure 2A shows the competition scenario described for the two established clonotypes, where the size of each ${\mathcal {Q}}_{i}$ circle represents the magnitude of $\varphi _{i}$, and the colour of each region represents the magnitude of $\nu _{ij}^{k}$, with a darker colour indicating a greater value. In this competition scenario the values of $p_{2,1}$ and $\nu _{2,1}$ were chosen to be 1/3 and 1, respectively.

Making use of the method described in Appendix G (see Supplementary Material), we calculated the mean time to extinction for all initial states with at most $10^2$ total cells and found them to range between 80 to 125 years. Given these long extinction times it was appropriate to approximate the QSD of this competition process. In Fig. 2B we plot the marginal distribution for clonotype 2. We show this distribution for only one of the clonotypes, since the competition between them is symmetric, and thus clonotypes 2 and 3 are identical before the introduction of clonotype 1. We observe that for the competition scenario considered, with most of the self-pMHCs in the hard niche, the approximation ${\mathcal {X}}^{(1)}$ better describes the behaviour of the system (see Gillespie simulations in Fig. 2B). However, we note that shifting ${\mathcal {X}}^{(2)}$ by one cell results again in a good approximation. This is to be expected for these small population sizes and for the hypothesis of an immortal cell in this process.

**Figure 3:**
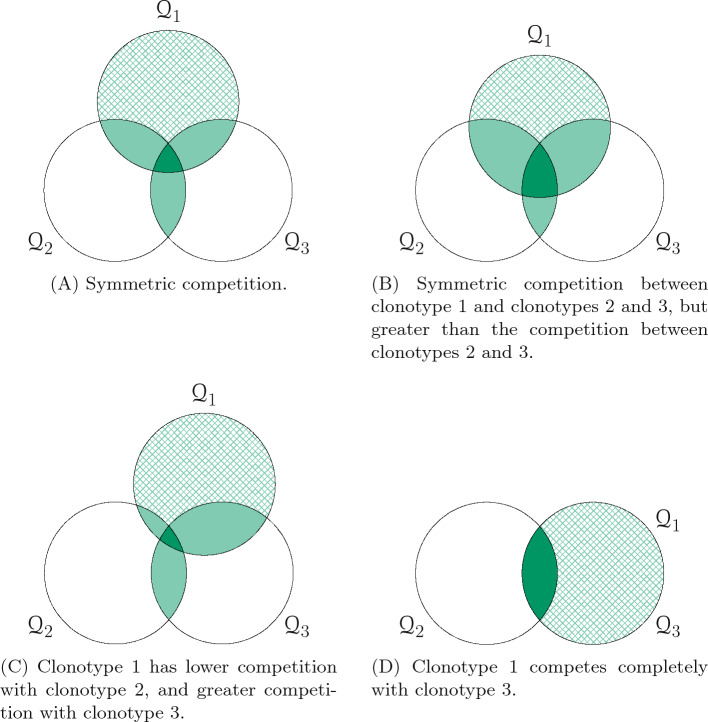
Competition scenarios when a new clonotype is introduced in a two-dimensional clonotype system. Shaded areas represent sets of self-pMHCs that are in the soft niche, with a darker shade meaning a lager value of the mean niche overlap ($\nu _{ij}^{k}$). Cross-hatched areas represent regions where we consider both the soft and hard niche cases.

Once clonotype 1 enters the periphery we will consider four different competition scenarios across a spectrum of symmetries, ranging from full symmetry to complete asymmetry. In the first scenario, Fig. 3A, we assume all clonotypes to be competing symmetrically; that is, all one-on-one competitions have the same probability. For the second scenario, Fig. 3B, we hypothesise symmetric competition between clonotype 1 and clonotypes 2 and 3, but these competitions are greater than the competition between clonotypes 2 and 3, leaving the new clonotype at a disadvantage. Our third scenario, Fig. 3C, has clonotype 1 competing more for homeostatic stimuli with clonotype 3 and less with clonotype 2, giving clonotype 2 an advantage. Our final scenario, Fig. 3D, represents the case of extreme asymmetry in which clonotypes 1 and 3 compete completely for stimuli. In all scenarios, we consider self-pMHCs recognised by more than one clonotype to be in the soft niche, with the value of the mean niche overlap increasing as the number of clonotypes increases. For the self-pMHCs recognised only by clonotype 1, we consider both hard and soft niches.

The diagrams in Fig. 3 were used as a guide to choose the probabilities shown in Table 1. Since we are considering three different clonotypes, we can use Eq. (7) to determine all the probabilities with only the four values given in Table 1. In the case of a single clonotype with no direct competitors ($\eta =1$), the mean time to extinction for mean niche overlap values greater than 10 is negligible^8^. Therefore, the values chosen for the mean niche overlap in our competition scenarios were: 1 for self-pMHCs recognised by two clonotypes, and 10 for self-pMHCs recognised by three clonotypes. Since we are considering clonotypes 2 and 3 to be homeostatically established, their initial state will be the mean of the QSD for the competition between them rounded to the nearest integer, which with the chosen parameters is the state (8, 8) (see Fig. 2B).

**Table 1:** Parameters for the competition scenarios shown in Fig. 3. The base value considered for $\varphi$ is 10, and we also consider the following values: $\varphi = 1, 10^{2}$.

	(a)	(b)	(c)	(d)
$p_{1,1}^{1}$	2/9	1/3	1/9	0
$p_{1,1}^{2}$	2/9	1/3	5/9	2/3
$p_{1,2}^{1}$	1/9	1/9	1/9	1/3
$p_{2,1}^{2}$	2/9	2/9	2/9	0
$\nu _{i,1}^{k}$	1 clonotype^8^			
$\nu _{i,2}^{1}$	10 clonotypes^8^			
$\varphi _{i}$	$1,10,10^{2}\text { cells}\cdot \text {year}^{-1}$^20^			
$\mu _{i}$	$1\text { year}^{-1}$			
$\langle n\rangle$	$10\text { cells}$			

### Clonal distributions at the first extinction event

**Figure 4:**
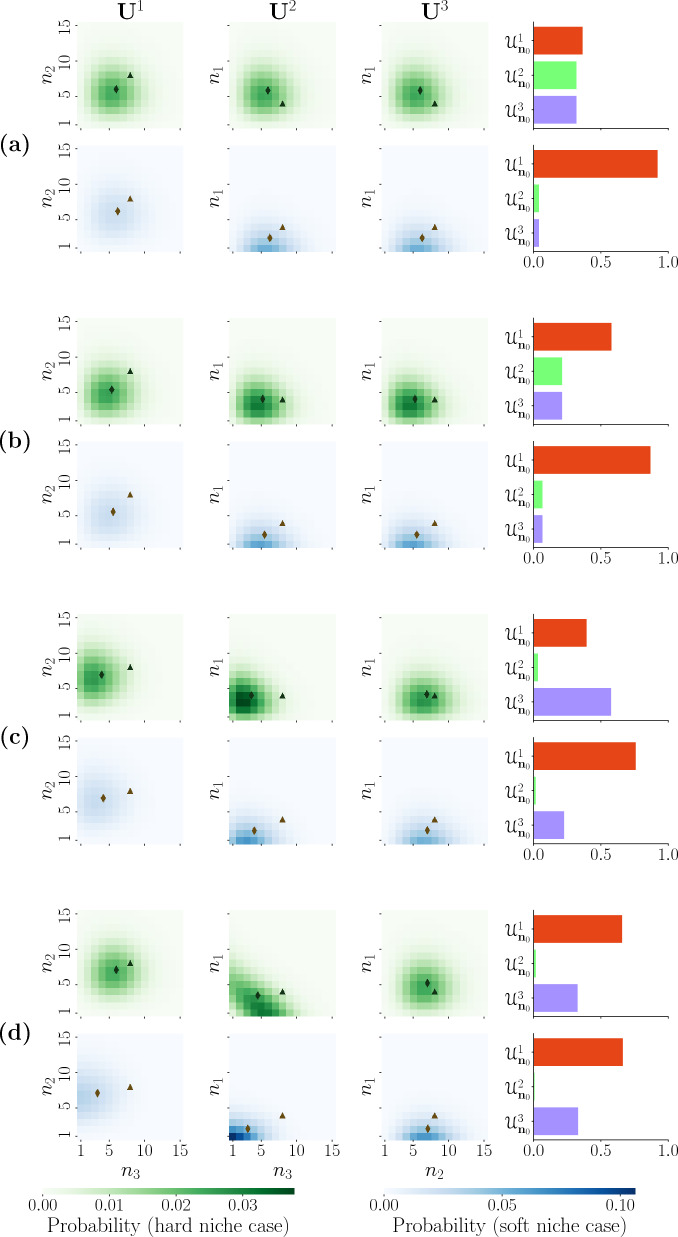
Distributions of clonal sizes at the time of the first extinction event (each column, ${\textbf{U}}^{i}$ for $i=1,2,3$, represents the extinction of clonotype *i* in the hard and soft niche cases (clonotype 1), green and blue, respectively, with initial state ${\textbf{n}}_{0}=(4,8,8)$ using the method described in Appendix H in the Supplementary Material). The fourth column shows the probability for each clonotype to be the first becoming extinct, ${\mathcal {U}}^{i}_{\mathbf {n_{0}}}$. Each panel (**a**, **b**, **c** and **d**) corresponds to one of the competition scenarios shown in Fig. 3. A triangle represents the initial state and a diamond indicates the mean of the resulting distribution of clonal sizes at the time of the first extinction event.

Using the method described in Appendix H in the Supplementary Material, we calculate the distribution of clonal sizes at the time of the first extinction event for the four different scenarios, and with the new clonotype in the hard or soft niche. Figure 4 shows the distribution of clonal sizes with an initial state, ${\textbf{n}}_{0}=(4,8,8)$, as well as the probability for each clonotype to be the first to become extinct, ${\mathcal {U}}^{i}_{{\textbf{n}}_{0}}$. A triangle represents the initial state considered, ${\textbf{n}}_{0}=(4,8,8)$, and a diamond represents the mean of the resulting distribution of clonal sizes at the time of the first extinction event.

Our results indicate that the probability of the new clonotype (clonotype 1) being the first to become extinct drastically increases when we compare the hard and soft niche cases in all the scenarios except (d). This shows that if the clonotype introduced is in the soft niche, its probability of extinction is greater, putting it at a large disadvantage against the other two homeostatically established clonotypes. In scenario (d) however, we see that not only does the probability of extinction of clonotype 1 change, but both ${\mathcal {U}}^{1}_{{\textbf{n}}_{0}}$ and ${\mathcal {U}}^{3}_{{\textbf{n}}_{0}}$ see a marginal increase. This different behaviour can be explained given that for (d) not only a new clonotype is being introduced in the system, but clonotype 3 changes from hard to soft niche, since this is a quality of the set of self-pMHCs and not of the clonotype itself. This implies that while in scenario (d) clonotype 1 sees no significant change in its extinction probability between the hard and soft niche cases, if it exits the thymus with more clonotypes that will also compete with it, this will reduce the advantage of clonotypes it competes with. By comparing the probabilities of extinction for clonotype 1 in the four different scenarios, we observe that in the hard niche case the most favourable scenario for its survival is scenario (a), of symmetric competition. This is the scenario in which it has less competition with the other two clonotypes overall. On the other hand, when we consider the soft niche case, we see that the most favourable scenario is scenario (d), in which the new clonotype competes completely for stimuli with an established clonotype. One likely explanation of this seemingly counter-intuitive behaviour is that, in scenario (d) not only is clonotype 1 exerting pressure on the established clonotype, but other clonotypes not explicitly modelled are doing so too, implying that the competitive pressure from clonotypes in ${\mathcal {M}}$ is shared with between clonotypes 1 and 3.

If we focus on the cases when clonotype 1 is not the first to become extinct, we see that in scenario (a) we expect its size to have rebounded into an established state in which both surviving clonotypes have fewer cells than the mean of the QSD of two competing clonotypes (before clonotype 1 was introduced). As a result the new clonotype expanded, while the established one contracted. In the soft niche we see that the size of the new clonotype does not bounce back to a homeostatic state, but instead both surviving clonotypes see a reduction in their number of cells. In scenario (b) for the hard niche case, we again see a move to a homeostatic state. However, there is little change in the size of the new clonotype, and a decrease in the size of the established clonotype to match the population of the surviving one. In the soft niche for this scenario we see a similar behaviour coupled with a reduction in the population size of clonotype 1. In scenario (c) we see a break from the symmetry observed between ${\textbf{U}}^{2}_{{\textbf{n}}_{0}}$ and ${\textbf{U}}^{3}_{{\textbf{n}}_{0}}$ in the previous scenarios, since the competition considered is no longer symmetric. If clonotype 2 is the first to become extinct, we expect the population of clonotype 3 (which has a greater competition with clonotype 1) to decrease until it matches that of clonotype 1. On the other hand, if clonotype 3 becomes extinct first we see very little change in the population of clonotype 2 (which has a lower competition with clonotype 1), and only a minor change in the size of clonotype 1. Lastly, in scenario (d) we see an interesting change in the shape of the clonal size distribution when clonotype 2 becomes extinct first. Since clonotypes 1 and 3 directly compete for all stimuli, they behave as a single population. Thus, the distribution is no longer centred around a point, but around a line where the sum of both populations is constant. However given the uneven initial state, the distribution has more density on the end that has clonotype 3 surviving with more cells than clonotype 1. If clonotype 3 is the first to become extinct, we see the same behaviour observed before where the population of the larger surviving clonotype decreases to match that of the new clonotype. In the soft niche we see an interesting behaviour when clonotype 2 is the first to become extinct. Since both remaining clonotypes are in the soft niche, the distribution has most of its density accumulated around state (1, 1), implying that even if these two clonotypes survive they can very easily become extinct due to their small population sizes.

**Figure 5:**
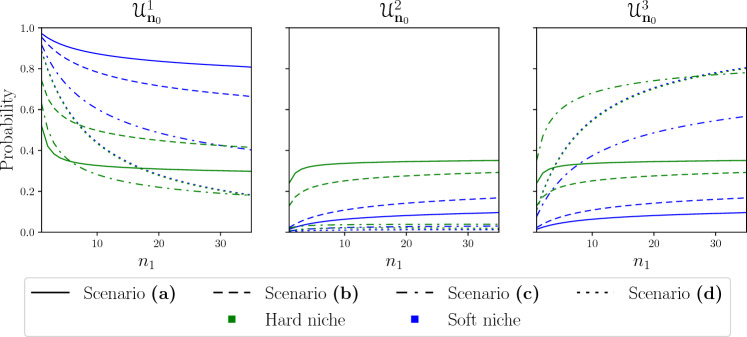
Probability of extinction for each clonotype, ${\mathcal {U}}^{i}_{{\textbf{n}}_{0}}$, in the four scenarios with hard and soft niche cases (for clonotype 1) as a function of the initial number of cells in clonotype 1, calculated using the method described in Appendix H in the Supplementary Material. The initial number of cells of the other two clonotypes is the mean of the QSD of their two-dimensional competition process, namely $(n_{2}, n_{3})=(8,8)$.

We now turn our focus to the ${\textbf{U}}^{1}$ distributions. We can see that even when the new clonotype is the first to become extinct, it has a negative effect on the populations of the established clonotypes, reducing their average sizes in all cases. To better understand the effect the new clonotype has on the homeostatically established clonotypes, we calculate the probability for each clonotype to become extinct as a function of the initial number of cells in the new clonotype and plotted them in Fig. 5.

The first thing we observe is that when comparing the hard and soft niche cases (clonotype 1) in each scenario, the probability of clonotype 1 becoming extinct first is always higher in the soft niche than in the hard niche case. For clonotypes 2 and 3 we see the opposite behaviour, with these clonotypes being more likely to become extinct first in the hard niche case. The only exception to this (by a minimal margin) is clonotype 3 in scenario (d), since in this case clonotype 3 has the added disadvantage of being in the soft niche, which increases its probability to become extinct. Another property we observe of the probability of extinction is that it very quickly becomes saturated; that is, it shows little sensitivity to changes in the number of initial cells in the new clonotype after a certain value (or threshold). The threshold level depends on the competition scenario considered. This implies that even a new clonotype with a small initial population can perturb the two-dimensional system and make one of the established clonotypes more likely to become extinct first. The largest difference between hard and soft niche cases is seen in scenario (a) for clonotype 1, where the probability of clonotype 1 being the first to become extinct is almost halved when comparing the soft to the hard niche. This is due to the fact that in this case the value of $p_{1,1}^{1}$ is highest, meaning that the proportion of self-pMHCs shared with clonotypes 2 and 3 is the lowest. Thus, a change in the mean niche overlap has a very strong effect on clonotype 1, since it changes from a scenario of low competition to one of complete competition. This is the same reason why we see such little change in scenario (d), since clonotype 1 is already competing for all of its stimuli and a change in the mean niche overlap has a much weaker effect. From this figure we also learn that in the hard niche case there are different optimal competition scenarios for the survival of the new clonotype. For initial numbers of cells fewer than six, we find the lowest probability of extinction in the symmetric competition scenario (a). For values between 6 and 30 the optimal competition is the asymmetrical competition scenario (b). Finally, for values above 30 complete competition with an established clonotype gives clonotype 1 the lowest probability of becoming extinct first. Interestingly, this behaviour is completely lost in the soft niche case, where we see that there is only one optimal competition scenario for all possible initial numbers of cells, namely complete competition with another clonotype (scenario (d)).

**Figure 6:**
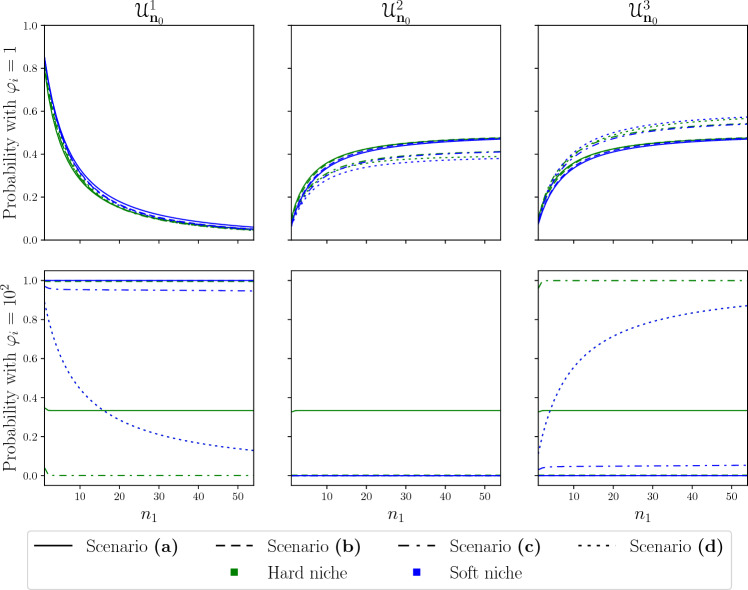
Probability of extinction for each clonotype in the four scenarios for the hard and soft niche cases (for clonotype 1) as a function of the initial number of cells in clonotype 1 for $\varphi =1,10^2$ (top and bottom row, respectively). The probabilities were calculated using the method described in Appendix H in the Supplementary Material. The initial number of cells in the other two clonotypes is the same as in Fig. 5, namely $(n_{2}, n_{3})=(8,8)$.

We also considered the effect of increasing and decreasing the total amount of stimulus available, $\varphi$, by an order of magnitude. In Fig. 6 we have plotted the probabilities of extinction, ${\mathcal {U}}^{i}_{{\textbf{n}}_{0}}$, for the case when $\varphi =1,10^2$. For $\varphi =1$ we see that, due to the scarcity of stimulus, there is little effect from the competition. Yet in the hard niche we still see smaller probabilities of extinction for clonotype 1, and higher probabilities for the established clonotypes. The strongest effect on the extinction probabilities in this case comes from the initial number of cells. However, in contrast to Fig. 5 the probabilities do not become saturated as quickly, meaning that in this case each cell of clonotype 1 has a weaker effect on the sizes of the established clonotypes. For $\varphi =10^2$ we see the opposite behaviour, where the probabilities become saturated so quickly that they appear as almost completely horizontal lines, with the notable exception of scenario (d). The first thing we note is that for clonotype 2 all the probabilities are negligible, except for scenario (a) in the hard niche case. In this case, given the overabundance of stimulus, the competition between clonotypes has no effect and all clonotypes are equally likely to be the first to become extinct. In scenario (b), for both hard and soft niche cases, the increased competition causes the extinction probability to accumulate on clonotype 1. The asymmetric competition of scenario (c) accumulates most of the probability of extinction on clonotype 1 and 3 in the soft and hard niche cases, respectively. Finally, in scenario (d) we observe a similar behaviour to that with $\varphi =1,10$, where the probabilities of clonotypes 1 and 3 are coupled and the difference between hard and soft niche is minimal. This behaviour stems from the fact that we have complete competition for stimuli, and regardless of its abundance the competitive exclusion principle^34^ tells us that one population must become extinct. With every other aspect of the populations being equal, this means that initial conditions completely determine which clonotype is most likely to become extinct.

### Number of divisions before extinction of a clonotype

**Figure 7:**
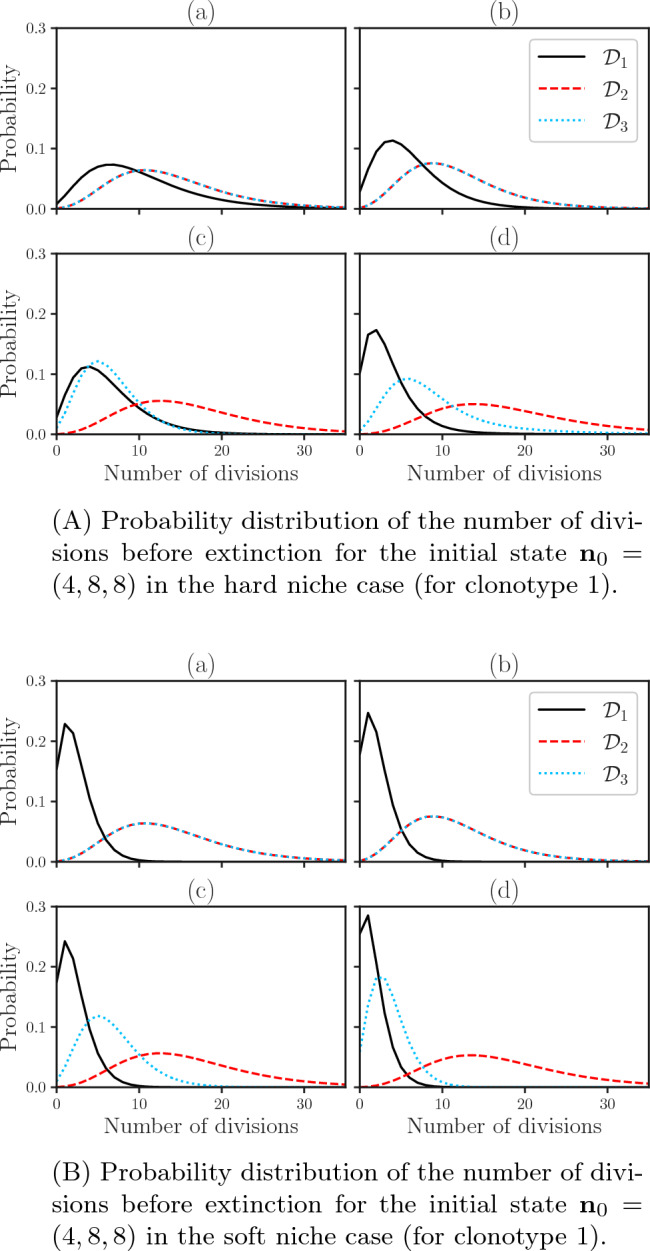
Probability distribution of the number of divisions before extinction for the initial state ${\textbf{n}}_{0}=(4,8,8)$ calculated using the method described in Appendix J (see Supplementary Material). (**A**) shows the distributions when clonotype 1 is in the hard niche case and (**B**) in the soft niche case.

We now calculate the probability distribution of the number of divisions before extinction of each clonotype, ${\mathcal {D}}_{i}$, making use of the method described in Appendix J (see Supplementary Material). Figure 7 shows these distribution for the four scenarios in the hard and soft niche cases (for clonotype 1) for the initial state ${\textbf{n}}_{0}=(4,8,8)$.

We can see that for scenarios (a) and (b), in both the hard and soft niche cases (for clonotype 1), the division distributions for clonotypes 2 and 3 remain mostly unchanged since they are not only competing in the same way but have the same initial number of cells. In scenario (c), we observe the distribution for clonotype 3 shifted to the right when compared to clonotype 2, due to the increase in competition for clonotype 3, but again there is no notable difference between the hard and soft niche cases. We see a break in these similarities between the hard and soft niche cases in scenario (d). Here, the distribution of divisions of clonotype 3 is narrower and centred around smaller values in the soft niche case when compared to the hard niche case. This is due to the fact that the new clonotype being in the soft niche changes all self-pMHCs recognised by clonotype 3 from the hard to the soft niche, greatly reducing its probability of dividing a greater number of times before becoming extinct. If one focuses on the new clonotype (clonotype 1), one observes that regardless of the niche considered in scenarios (a) and (b), the median of the number of divisions is lower than the other modelled clonotypes, with this difference being greater in the soft niche case. In scenario (a), this behaviour is caused by the smaller population size of the new clonotype (since every other aspect of the competition is symmetric). The behaviour is amplified in scenario (b) given the increased competition experienced by the new clonotype. In scenario (c), we see that in the hard niche case the distribution of divisions of the new clonotype is very similar to that of clonotype 3. This is due to the change in the distribution for clonotype 3 due to its increased competition. However, this similarity is undone in the soft niche case where the distribution for the new clonotype is narrower and has a smaller median. This agrees with our previous observation in Fig. 5: the niche in which the new clonotype is has a bigger impact on its fate than its competition scenario. Finally, in scenario (d) we see that in the hard niche case the distribution already has a low median and is rather narrow. This behaviour is stronger in the soft niche case. In the hard niche case, scenario (d) has the lowest median and the most narrow distribution for the new clonotype overall, followed by the distributions of scenarios (b), (c), and (a), in that order. This agrees with the probabilities of extinction for the initial state ${\textbf{n}}=(4,8,8)$ in Fig. 5, where we see that these probabilities decrease in the same order. In the soft niche case we can see a direct relation between the probabilities of extinction and distribution of divisions for scenarios (a), (b), and (c), but not for scenario (d). This can be justified since the probabilities of extinction are changing due to an increased chance of clonotype 3 becoming extinct and not a direct change to the extinction of the new clonotype.

## Discussion

Maintaining the diversity of the T cell repertoire is essential for the immune system to mount a strong and effective immune response^35–38^. Promoting survival of significantly different clonotypes maximises this diversity by allowing TCRs with similar self-pMHC recognition profiles to become extinct. However, it has been observed that several clonotypes often overlap on their recognition profile (e.g.^25,26,39^) as a compromise between TCR diversity and coverage over the space of foreign antigens. This motivates the multi-variate representation we have developed in this manuscript, which extends and generalises that presented in^17^ to three or more clonotypes.

We showed that the cellular division rate of competing clonotypes decreases as the overlap in their recognition profiles increases. That is, we see the effects of competitive exclusion as the distribution of the number of divisions shows a decrease in its mode (the peak moves to the left), when there is an increase in the competition for self-pMHCs. Furthermore, we mathematically showed that extinction of all clonotypes is certain; that is, given sufficient time (which can be larger than the biological timescales) the populations of all clonotypes will become extinct. The mean time to this extinction event is bounded, and it depends on the mean number of cells per clonotype. A feature of the proposed competition process, prior to the first extinction event, is that the system is driven to a state where all clones have low numbers of cells, with the specific number of cells depending on their recognition overlap. This agrees with the biological assumption that in a homeostatic state each clonotype consists of only a few cells, with the exact number varying from clonotype to clonotype^20,40^.

Our multi-variate competition model shows that the introduction of a new clonotype to the periphery perturbs the homeostatically established clonotypes. We see this in the decreased mean number of cells after the first extinction event when compared to the initial state of the process. Even in the scenario with the least competition for stimuli (scenario (a)) we see a perturbation of the established clonotypes. More than this, the probability of extinction as a function of the initial number of cells in the new clonotype very quickly becomes saturated (see Fig. 5). This implies that even a clonotype with a low number of initial cells has great potential to perturb already established clonotypes.

From the distribution of divisions before extinction, we can say that in the soft niche case we always expect the new clonotype to experience very little proliferation before becoming extinct. Even in scenario (a), where competition with other modelled clonotypes is the lowest, we see a distribution of divisions that is very narrow and centred around a small value. This major change outlines an important feature of the soft niche; even when considering a mean niche overlap of only ten clonotypes, the probability of extinction is greatly increased and the distribution of divisions is made more narrow and moved to the left. This major change in behaviour between the hard and soft niche cases can be interpreted in two ways: *(i)* for the soft niche assumption to be correct in the naive T cell repertoire, the mean niche overlap value must be a small number of clonotypes ($1<\nu _{ij}^{k}<10$), otherwise a new clonotype entering the periphery would have a very low probability of becoming established, and the naive T cell repertoire would become mostly static; *(ii)* if the mean niche overlap is not small, then T cells in the soft niche must have a very fast turnover, and make up for most of the thymic output to counteract this low probability of proliferation and establishment. These two interpretations are made under the assumption that the thymic output is mostly homogeneous; that is, we assume that most of the cells exiting the thymus are either part of the hard or soft niche. By relaxing this assumption and considering a heterogeneous thymic output, instead of a homogenous one, we can think of the naive T cell repertoire as being divided in two sets: one set comprised of clonotypes in the hard niche, which is deeply established (with low extinction probabilities) and is expected to remain mostly constant through life, and another set of clonotypes in the soft niche, which is constantly changing and is more dynamic through life (as clonotypes appear from the thymus and become extinct due to clonal competition). This type of heterogeneity would allow for the maintenance of a T cell repertoire that has the capability of constantly producing new clonotypes (in the soft niche), while maintaining other clonotypes (in the hard niche) throughout life.

So far we have only discussed the possible effects of a distribution of soft and hard niche clonotypes exiting the thymus, assumed to be constant throughout the ageing process^37,41,42^. However, given the known changes in thymic output through life, such as thymic involution^43,44^, and other age-related changes^45^, it is plausible that the composition of thymic emigrants (in terms of hard and soft niche, or clonal size) changes as well during the lifespan of a host. If we consider thus, not a constant mixture of hard and soft niche clonotypes exiting the thymus, but a mixture that evolves from mostly hard niche clonotypes during fetal stages to mostly soft niche clonotypes during adulthood, then our model predicts the early establishment of clonotypes that exited the thymus early in life, and then a declining supply of mostly short-lived clonotypes later in life. This decline in the production of soft niche clonotypes could be justified by the fact that during the initial development of the T cell repertoire, we naturally expect there to be fewer clonotypes in the periphery, and therefore smaller values of the mean niche overlap $\nu _{ij}^{k}$. Furthermore, this behaviour is compatible with the analysis by Gaimann et al.^46^, in which T cell clonotype sizes were found to follow a power-law distribution, where clonotypes generated during the early fetal stages (characterised by no nucleotide insertions during V(D)J recombination^47^) were found to be the most enriched in the periphery. A dynamical analysis, making use of the competition model proposed here, of clonotype diversity, sizes and niches, with parameters corresponding to a human or murine host, may be feasible.

### Supplementary Information

Supplementary Information.

## Data Availability

All data generated or analysed during this study are included in this published article [and its supplementary information files].

## Code availability

The python codes used to generate the figures in section “Results” have been made available at https://doi.org/10.5281/zenodo.6342372^48^.
