# Supplementary material for: Multi-variate model of T cell clonotype competition and homeostasis
Source: Sci Rep. 2023 Dec 11;13:21995. doi: 10.1038/s41598-023-46637-4 (PMC10713556; doi:10.1038/s41598-023-46637-4)
Supplement: Supplementary file 1 — Supplementary Information. [file 41598_2023_46637_MOESM1_ESM.pdf]

Appendix A

General formulation of the transition rates

We first write Eq. (1) making use of the following definition. n_{iq} is the number of cells that are not of clonotype i and receive stimuli from self-pMHC q; that is, $n_{iq} = n_q - n_i$, so that Eq. (1) becomes

$$\Lambda^{(i)}(\mathbf{n}) = \sum_{q \in \mathcal{Q}_i} \frac{\gamma}{n_i + n_{iq}}. \tag{A.1}$$

By writing the birth rate in this manner it is easy to see that it depends not only on clonotype i, but on all other clonotypes which compete for stimuli from self-pMHCs in $\mathcal{Q}_i$.

For a fixed clonotype i, let us define $\mathcal{C}_i$, the set of all clonotypes in $\mathcal{C}$ except for i, $\mathcal{C}_i := \mathcal{C} \setminus \{i\}$. Let us consider the subsets I_{ij} of the power set, $\mathcal{P}(\mathcal{C}_i)$, which consist of all elements with cardinality $j = 0, 1, 2, \ldots, \eta - 1$; that is, I_{ij} contains all the possible subsets of $\mathcal{C}$ with j elements which do not contain clonotype i. We define I_{ij}^k as the k-th element of I_{ij} under the lexicographical order, for $k = 1, 2, \ldots, \binom{\eta-1}{j}$, since we note that $|I_{ij}| = \binom{\eta-1}{j}$. Altogether, this means that $I_{i,j}^k$ represents the k-th set under the lexicographical order of j clonotypes in $\mathcal{C}_i$. As an illustrative example consider the set of clonotypes $\mathcal{C} = \{1, 2, 3, 4, 5\}$, and $j = 3$, then the sets in $I_{3,3}$ are

$$\begin{aligned} I_{i,3} &= \left\{ I_{3,3}^1, I_{3,3}^2, I_{3,3}^3, I_{3,3}^4 \right\} \\ &= \Big\{ \{1,2,4\}, \{1,2,5\}, \{1,4,5\}, \{2,4,5\} \Big\}. \end{aligned} \tag{A.2}$$

The sets described in this example are shown in Figure A.1.

These sets allow us to partition $\mathcal{Q}_i$, the set of self-pMHCs that stimulate clonotype i, into sets, $\mathcal{Q}_{ij}^k$, of self-pMHCs that stimulate precisely clonotype i and those in I_{ij}^k. These $\mathcal{Q}_{ij}^k$ sets are defined as follows

$$\mathcal{Q}_{ij}^k = \underbrace{\mathcal{Q}_i}_{\substack{\text{stimulates} \\ \text{clonotype } i}} \cap \underbrace{\left(\bigcap_{l \in I_{ij}^k} \mathcal{Q}_l \right)}_{\substack{\text{stimulates all} \\ \text{clonotypes in } I_{ij}^k}} \cap \underbrace{\left(\bigcap_{l \in \overline{I_{ij}^k}} \overline{\mathcal{Q}_l} \right)}_{\substack{\text{does not stimulate} \\ \text{any other clonotypes}}} , \tag{A.3}$$

where $\overline{I_{ij}^k}$ is the complement of I_{ij}^k in $\mathcal{C}_i$ and $\overline{\mathcal{Q}_i}$ is the complement of $\mathcal{Q}_i$ in $\mathcal{Q}$. By

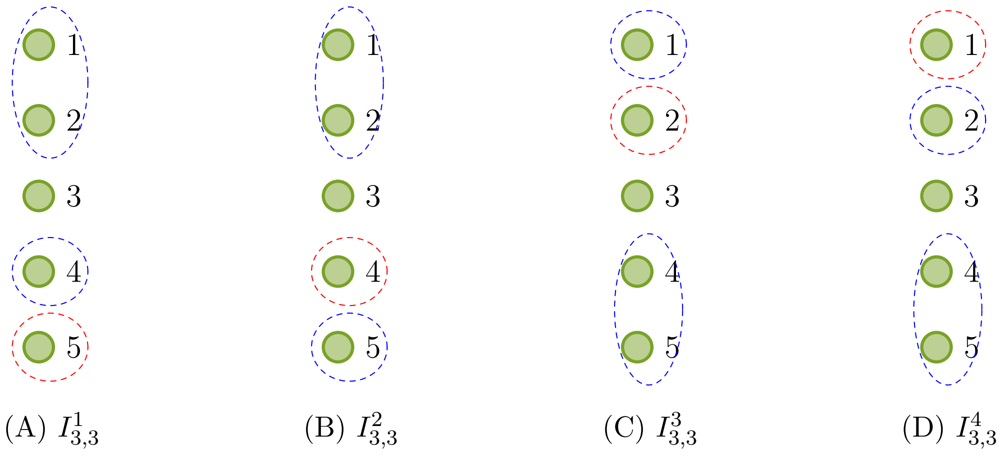

Figure A.1: Representation of the sets in $I_{3,3}$ (blue) and their complement, $\overline{I^k_{3,3}}$, in $\mathcal{C}_3$ (red), for the case $\mathcal{C} = \{1, 2, 3, 4, 5\}$. (A), (B), (C), and (D) correspond to the sets defined in Eq. (A.2).

construction the sets $\mathcal{Q}^k_{ij}$ are disjoint and their union is $\mathcal{Q}_i$, so that we can write

$$\mathcal{Q}_i = \bigcup_{j=0}^{\eta-1} \bigcup_{k=1}^{\binom{\eta-1}{j}} \mathcal{Q}^k_{ij}. \tag{A.4}$$

With this partition we can now rewrite Eq. (A.1) as the sum of stimuli from self-pMHCs that are shared with $j = 0, 1, \ldots, \eta - 1$ other clonotypes. In this way, we get a better understanding of the competition for self-pMHCs between the η different clonotypes. We have

$$\Lambda^{(i)}(\mathbf{n}) = \gamma \sum_{j=0}^{\eta-1} \sum_{k=1}^{\binom{\eta-1}{j}} \sum_{q\in\mathcal{Q}^k_{ij}} \Lambda^k_{ijq}(\mathbf{n}), \tag{A.5}$$

where $\Lambda^k_{ijq}(\mathbf{n})$ is the fraction of stimulus provided to clonotype i by a self-pMHC q that it shares only with clonotypes in I^k_{ij}. We can then write

$$\Lambda^k_{ijq}(\mathbf{n}) = \frac{1}{n_i + \sum\limits_{l\in I^k_{ij}} n_l + n^k_{ijq}},$$

with n^k_{ijq} defined as the number of cells not in $\mathcal{C}$ that receive stimulus from $q \in \mathcal{Q}^k_{ij}$; that is, $n^k_{ijq} = n_q - n_i - \sum_{l\in I^k_{ij}} n_l$. The product of $\Lambda^k_{ijq}(\mathbf{n})$ and γ is the homeostatic proliferation stimulus provided to clonotype i by self-pMHC q, which also stimulates all clonotypes in I^k_{ij}. We can now subdivide the $\mathcal{Q}^k_{ij}$ sets further by considering the number of clonotypes not in $\mathcal{C}$ that can receive stimulus from $q \in \mathcal{Q}^k_{ij}$. Let $\mathcal{Q}^k_{ijr}$ with $r = 0, 1, \ldots, M$ denote the set of self-pMHCs that stimulate clonotype i, the clonotypes in I^k_{ij}, and r other clonotypes in $\mathcal{M}$. With these sets we can rewrite Eq. (A.5) as follows

$$\Lambda^{(i)}(\mathbf{n}) = \gamma \sum_{j=0}^{\eta-1} \sum_{k=1}^{\binom{\eta-1}{j}} \sum_{r=0}^{M} \sum_{q\in\mathcal{Q}^k_{ijr}} \Lambda^k_{ijq}(\mathbf{n}). \tag{A.6}$$

To simplify Eq. (A.6) and for practical purposes, we will make a mean field approximation, as discussed in [11]. Let us define the following quantities, average and variance of n_{ijq}^k over the set of $q \in \mathcal{Q}_{ijr}^k$:

$$\mathbb{E}_{ijr}^k\left[n_{ijq}^k\right] := \frac{1}{|\mathcal{Q}_{ijr}^k|} \sum_{\ell \in \mathcal{Q}_{ijr}^k} n_{ij\ell}^k, \tag{A.7a}$$

$$\mathbb{V}_{ijr}^k\left[n_{ijq}^k\right] := \frac{1}{|\mathcal{Q}_{ijr}^k|} \sum_{\ell \in \mathcal{Q}_{ijr}^k} \left(n_{ij\ell}^k - \mathbb{E}_{ijr}^k\left[n_{ijq}^k\right]\right)^2, \tag{A.7b}$$

respectively. Then, making use of Eq. (A.7a), Eq. (A.7b), the Taylor series of $\Lambda_{ijq}^k(\mathbf{n})$ as a function of n_{ijq}^k, and the properties of the expected value [2, Chapter 2], we rewrite the sum of $\Lambda_{ijq}^k(\mathbf{n})$ as follows

$$\sum_{q \in \mathcal{Q}_{ijr}^k} \Lambda_{ijq}^k(\mathbf{n}) = |\mathcal{Q}_{ijr}^k| \left(\frac{1}{n_i + \sum_{l \in I_{ij}^k} n_l + \mathbb{E}_{ijr}^k\left[n_{ijq}^k\right]} + \frac{\mathbb{V}_{ijr}^k\left[n_{ijq}^k\right]}{\left(n_i + \sum_{l \in I_{ij}^k} n_l + \mathbb{E}_{ijr}^k\left[n_{ijq}^k\right]\right)^3} + \cdots \right). \tag{A.8}$$

Our first assumption is that the first term in Eq. (A.8) dominates the sum. This assumption stems from the fact that in carrying out the mean field approximation, $\mathbb{V}_{ijr}^k\left[n_{ijq}^k\right]$ is considered to be small. Since it appears as a factor of the second and subsequent terms, we consider them to be small as well. The second assumption is that given r, the average number of cells per clonotype in $\mathcal{M}$ competing with i and all clonotypes in I_{ij}^k for stimuli from self-pMHC $q \in \mathcal{Q}_{ijr}^k$ is the same as the average clonotype size; that is

$$\mathbb{E}_{ijr}^k\left[n_{ijq}^k\right] = r\langle n \rangle, \tag{A.9}$$

where $\langle n \rangle$ is the average clonotype size in $\mathcal{M}$ [11]. From Eq. (A.9) and our assumption in Eq. (A.8) we can write

$$\sum_{q \in \mathcal{Q}_{ijr}^k} \Lambda_{ijq}^k(\mathbf{n}) \approx \frac{|\mathcal{Q}_{ijr}^k|}{n_i + \sum_{l \in I_{ij}^k} n_l + r\langle n \rangle}. \tag{A.10}$$

The next step is to find an expression for $|\mathcal{Q}_{ijr}^k|$. In order to do so, we first need to find one for $|\mathcal{Q}_{ij}^k|$. Let p_{ij}^k denote the probability that a randomly chosen self-pMHC in $\mathcal{Q}_i$ will provide stimuli to all clonotypes in I_{ij}^k. This probability can be defined in terms of the cardinality of the $\mathcal{Q}_{ij}^k$ sets as follows

$$p_{ij}^k = \frac{|\mathcal{Q}_{ij}^k|}{|\mathcal{Q}_i|},$$

and from Eq. (A.4) we have

$$\sum_{j=0}^{\eta-1}\sum_{k=1}^{\binom{\eta-1}{j}} p_{ij}^k = 1.$$

Let us now consider the probability $p_{\cdot|ijk}$ that a clonotype in $\mathcal{M}$ is stimulated by a self-pMHC in $\mathcal{Q}_{ij}^k$. That is, any clonotype chosen at random from $\mathcal{M}$ is stimulated by a self-pMHC in $\mathcal{Q}_{ij}^k$, with probability $p_{\cdot|ijk}$. It is clear that the number of self-pMHCs in $\mathcal{Q}_{ijr}^k$ follows a binomial distribution, Binomial$(M, p_{\cdot|ijk})$, since it is the sum of M independent Bernoulli experiments, each with probability $p_{\cdot|ijk}$. Thus, we can write

$$|\mathcal{Q}_{ijr}^k| = |\mathcal{Q}_{ij}^k| \binom{M}{r} \left(p_{\cdot|ijk}\right)^r \left(1 - p_{\cdot|ijk}\right)^{M-r}. \tag{A.11}$$

Under the assumption that the number of modelled clonotypes is small compared to the total number of clonotypes, *i.e.*, $\eta \ll M$, we can use the Poisson approximation by introducing the parameter $\nu_{ij}^k = M p_{\cdot|ijk}$, so that we can write

$$|\mathcal{Q}_{ijr}^k| \approx p_{ij}^k |\mathcal{Q}_i| \frac{\left(\nu_{ij}^k\right)^r e^{-\nu_{ij}^k}}{r!}. \tag{A.12}$$

We call ν_{ij}^k the mean niche overlap for self-pMHCs that stimulate clonotype i and all clonotypes in I_{ij}^k [11]. Let $\varphi_i = \gamma|\mathcal{Q}_i|$, then from Eq. (A.6), Eq. (A.10), and Eq. (A.12) we obtain

$$\begin{aligned}\Lambda^{(i)}(\mathbf{n}) &\approx \gamma \sum_{j=0}^{\eta-1}\sum_{k=1}^{\binom{\eta-1}{j}}\sum_{r=0}^{M} p_{ij}^k |\mathcal{Q}_i| \frac{\left(\nu_{ij}^k\right)^r e^{-\nu_{ij}^k}}{r!} \frac{1}{n_i + \sum_{l \in I_{ij}^k} n_l + r\langle n\rangle} \\ &= \varphi_i \sum_{j=0}^{\eta-1}\sum_{k=1}^{\binom{\eta-1}{j}} p_{ij}^k e^{-\nu_{ij}^k} \sum_{r=0}^{M} \frac{\left(\nu_{ij}^k\right)^r}{r!} \frac{1}{n_i + \sum_{l \in I_{ij}^k} n_l + r\langle n\rangle}.\end{aligned} \tag{A.13}$$

We make use of this result to approximate the birth rate of clonotype i defined in Eq. (2) as follows

$$\lambda_{\mathbf{n}}^{(i)} = n_i \Lambda^{(i)}(\mathbf{n}) \approx \varphi_i n_i \sum_{j=0}^{\eta-1}\sum_{k=1}^{\binom{\eta-1}{j}} p_{ij}^k e^{-\nu_{ij}^k} \sum_{r=0}^{M} \frac{\left(\nu_{ij}^k\right)^r}{r!} \frac{1}{n_i + \sum_{l \in I_{ij}^k} n_l + r\langle n\rangle}. \tag{A.14}$$

Note that from the definition of φ_i we have

$$\gamma|\mathcal{Q}_{ij}^k| = \gamma p_{ij}^k |\mathcal{Q}_i| = p_{ij}^k \varphi_i.$$

Then, for any pair of clonotypes i, i' in $\mathcal{C}$ and a pair of sets I_{ij}^k, $I_{i'j'}^{k'}$, such that $I_{ij}^k \cup \{i\} = I_{i'j'}^{k'} \cup \{i'\}$, we have the following relation

$$\varphi_i p_{ij}^k = \varphi_{i'} p_{i'j'}^{k'}. \tag{A.15}$$

This constraint comes naturally from the fact that, while the sizes of $\mathcal{Q}_i$ and $\mathcal{Q}_{i'}$ can be different, the stimuli provided by $\mathcal{Q}_{ij}^k$ and $\mathcal{Q}_{i'j'}^{k'}$ is the same if $I_{ij}^k \cup \{i\} = I_{i'j'}^{k'} \cup \{i'\}$.

Furthermore, for any pair of clonotypes i, i' in $\mathcal{C}$, and a pair of sets I_{ij}^{k}, $I_{i'j'}^{k'}$, such that $I_{ij}^{k} \cup \{i\} = I_{i'j'}^{k'} \cup \{i'\}$, we also have the following constraint for their mean niche overlap parameters

$$\nu_{ij}^{k} = \nu_{i'j'}^{k'}. \tag{A.16}$$

The mean niche overlap is a characteristic of self-pMHCs and not of clonotypes, and therefore its value depends only on the sets $\mathcal{Q}_{ij}^{k}$ and $\mathcal{Q}_{i'j'}^{k'}$, and not on the specific clonotypes i and i'.

The birth and death rates of the process $\mathcal{X}$, which have been defined in Eq. (3) and Eq. (A.14), respectively, can be simplified for two limiting cases. The first one is that in which $\nu_{ij}^{k} \ll 1$ for all ν_{ij}^{k}. We call this the "hard niche" case, which is characterised by low competition with clonotypes in $\mathcal{M}$. In this case Eq. (A.14) simplifies to [11]

$$\lambda_{\mathbf{n}}^{(i)} = \varphi_i n_i \sum_{j=0}^{\eta-1} \sum_{k=1}^{\binom{\eta-1}{j}} \frac{p_{ij}^{k}}{n_i + \sum\limits_{l \in I_{ij}^{k}} n_l}. \tag{A.17}$$

The second case, when $\nu_{ij}^{k} \gg 1$ for all ν_{ij}^{k}, is called the "soft niche" case, where there is increased competition with clonotypes in $\mathcal{M}$. In this case we approximate Eq. (A.14) by [11]

$$\lambda_{\mathbf{n}}^{(i)} \approx \varphi_i n_i \sum_{j=0}^{\eta-1} \sum_{k=1}^{\binom{\eta-1}{j}} \frac{p_{ij}^{k}}{n_i + \sum\limits_{l \in I_{ij}^{k}} n_l + \nu_{ij}^{k} \langle n \rangle}. \tag{A.18}$$

Appendix B

Exact calculation of the QSD

First, we note that the probabilities defined in Eq. (10) follow the Kolmogorov differential equations

$$\frac{\mathrm{d}p_{\mathbf{n}}(t)}{\mathrm{d}t} = \sum_{i=1}^{\eta} \lambda^{(i)}_{\mathbf{n}^{(-i)}} p_{\mathbf{n}^{(-i)}}(t) + \sum_{i=1}^{\eta} \mu^{(i)}_{\mathbf{n}^{(+i)}} p_{\mathbf{n}^{(+i)}}(t) - \sum_{i=1}^{\eta} \left(\lambda^{(i)}_{\mathbf{n}} + \mu^{(i)}_{\mathbf{n}}\right) p_{\mathbf{n}}(t), \tag{B.1}$$

and that from Eq. (11) we can derive the following system of differential equations

$$\begin{aligned} \frac{\mathrm{d}q_{\mathbf{n}}(t)}{\mathrm{d}t} &= \frac{\mathrm{d}}{\mathrm{d}t}\frac{p_{\mathbf{n}}(t)}{p_{\overline{\mathcal{A}}}(t)} \\ &= \frac{1}{p_{\overline{\mathcal{A}}}(t)}\frac{\mathrm{d}p_{\mathbf{n}}(t)}{\mathrm{d}t} - \frac{q_{\mathbf{n}}(t)}{p_{\overline{\mathcal{A}}}(t)}\frac{\mathrm{d}p_{\overline{\mathcal{A}}}(t)}{\mathrm{d}t}. \end{aligned} \tag{B.2}$$

Then, we can use Eq. (B.1) to find an expression for Eq. (B.2) in terms of the birth and death rates of the competition process, $\lambda^{(i)}_{\mathbf{n}}$ and $\mu^{(i)}_{\mathbf{n}}$, as follows. For the first term of Eq. (B.2) we obtain

$$\begin{aligned} \frac{1}{p_{\overline{\mathcal{A}}}(t)}\frac{\mathrm{d}p_{\mathbf{n}}(t)}{\mathrm{d}t} &= \sum_{i=1}^{\eta} \lambda^{(i)}_{\mathbf{n}^{(-i)}} q_{\mathbf{n}^{(-i)}}(t) + \sum_{i=1}^{\eta} \mu^{(i)}_{\mathbf{n}^{(+i)}} q_{\mathbf{n}^{(+i)}}(t) \\ &\quad - \sum_{i=1}^{\eta} \left(\lambda^{(i)}_{\mathbf{n}} + \mu^{(i)}_{\mathbf{n}}\right) q_{\mathbf{n}}(t). \end{aligned} \tag{B.3}$$

For the second term of Eq. (B.2), we first use the law of total probability to rewrite $p_{\overline{\mathcal{A}}}(t)$ as

$$p_{\overline{\mathcal{A}}}(t) = 1 - \sum_{\mathbf{n}\in\mathcal{A}} p_{\mathbf{n}}(t). \tag{B.4}$$

However, since we are considering the competition process conditioned on non-extinction, our boundary is the set of states where only one clonotype has become extinct. Thus, when we calculate $\frac{\mathrm{d}p_{\overline{\mathcal{A}}}(t)}{\mathrm{d}t}$ the only relevant transitions are to states in which only one clonotype has become extinct. Let us, then, define $\mathcal{A}_i$ as the set of states where only clonotype i has gone extinct; that is, we write for $1 \leq i \leq \eta$

$$\mathcal{A}_i = \left\{\mathbf{n} \in \mathcal{S} : n_i = 0, n_k > 0 \quad \forall k \neq i \text{ with } 1 \leq k \leq \eta\right\}. \tag{B.5}$$

For the sake of computing $p_{\overline{\mathcal{A}}}(t)$, one can consider that states in $\mathcal{A}_i$ are absorbing for all $1 \leq i \leq \eta$, and then rewrite Eq. (B.4) as

$$p_{\overline{\mathcal{A}}}(t) = 1 - \sum_{i=1}^{\eta} \sum_{\mathbf{n} \in \mathcal{A}_i} p_{\mathbf{n}}(t).$$

If we take a derivative with respect to t, we can write

$$\frac{\mathrm{d}p_{\overline{\mathcal{A}}}(t)}{\mathrm{d}t} = -\sum_{i=1}^{\eta} \sum_{\mathbf{n} \in \mathcal{A}_i} \mu^{(i)}_{\mathbf{n}^{(+i)}} p_{\mathbf{n}^{(+i)}}(t). \tag{B.6}$$

We make use of Eq. (11) and Eq. (B.6) to show that the second term of Eq. (B.2) is

$$\frac{q_{\mathbf{n}}(t)}{p_{\overline{\mathcal{A}}}(t)} \frac{\mathrm{d}p_{\overline{\mathcal{A}}}(t)}{\mathrm{d}t} = -q_{\mathbf{n}}(t) \sum_{i=1}^{\eta} \sum_{\mathbf{m} \in \mathcal{A}_i} \mu^{(i)}_{\mathbf{m}^{(+i)}} q_{\mathbf{m}^{(+i)}}(t). \tag{B.7}$$

Note that we are only considering death events for clonotypes of size one. Then by Eq. (3) we can further simplify Eq. (B.7) to obtain

$$\frac{q_{\mathbf{n}}(t)}{p_{\overline{\mathcal{A}}}(t)} \frac{\mathrm{d}p_{\overline{\mathcal{A}}}(t)}{\mathrm{d}t} = -q_{\mathbf{n}}(t) \sum_{i=1}^{\eta} \sum_{\mathbf{m} \in \mathcal{A}_i} \mu_i q_{\mathbf{m}^{(+i)}}(t). \tag{B.8}$$

Substituting Eq. (B.3) and Eq. (B.8) in Eq. (B.2) we have

$$\begin{aligned} \frac{\mathrm{d}q_{\mathbf{n}}(t)}{\mathrm{d}t} = &\sum_{i=1}^{\eta} \lambda^{(i)}_{\mathbf{n}^{(-i)}} q_{\mathbf{n}^{(-i)}}(t) + \sum_{i=1}^{\eta} \mu^{(i)}_{\mathbf{n}^{(+i)}} q_{\mathbf{n}^{(+i)}}(t) \\ &- \sum_{i=1}^{\eta} \left(\lambda^{(i)}_{\mathbf{n}} + \mu^{(i)}_{\mathbf{n}}\right) q_{\mathbf{n}}(t) \\ &+ q_{\mathbf{n}}(t) \sum_{i=1}^{\eta} \sum_{\mathbf{m} \in \mathcal{A}_i} \mu_i q_{\mathbf{m}^{(+i)}}(t). \end{aligned}$$

The limiting conditional distribution (LCD) of the process is the limit as $t \to +\infty$ of $q_{\mathbf{n}}(t)$ [3]. If this limit exists, the resulting probability distribution is a QSD of the process, which in the case of a Markov process with finite state space is unique and therefore equal to the LCD [3]. Now, if there exists a probability distribution $\tilde{q}$ such that $\sum_{\mathbf{n} \in \mathcal{S} \setminus \mathcal{A}} \tilde{q}_{\mathbf{n}} = 1$, that also satisfies

$$\begin{aligned} 0 = &\sum_{i=1}^{\eta} \lambda^{(i)}_{\mathbf{n}^{(-i)}} \tilde{q}_{\mathbf{n}^{(-i)}} + \sum_{i=1}^{\eta} \mu^{(i)}_{\mathbf{n}^{(+i)}} \tilde{q}_{\mathbf{n}^{(+i)}} \\ &- \sum_{i=1}^{\eta} \left(\lambda^{(i)}_{\mathbf{n}} + \mu^{(i)}_{\mathbf{n}}\right) \tilde{q}_{\mathbf{n}} + \tilde{q}_{\mathbf{n}}(t) \sum_{i=1}^{\eta} \sum_{\mathbf{m} \in \mathcal{A}_i} \mu_i \tilde{q}_{\mathbf{m}^{(+i)}}(t), \end{aligned} \tag{B.9}$$

then, it is a QSD of the process. Finding an analytical solution of Eq. (B.9) is in general not possible, and thus, the QSD will be numerically approximated.

Appendix C

Position of states within levels with no extinction

For a given state $\mathbf{n} = (n_1, n_2, \ldots, n_\eta)$ in level $L^0(k)$, its position within the level under the colexicographical order is given by

$$\begin{aligned}
\mathrm{pos}_k(\mathbf{n}, \eta) =& \frac{1}{k-\eta}\left[\sum_{i=1}^{\eta}(n_i - 1)(k-\eta+1)^{i-1}\right. \\
&\left.+\sum_{i=3}^{\eta}(n_i - 1)(1-(k-\eta+1)^{i-1})\right] \\
&+\sum_{i=3}^{\eta}\left[\binom{k-1-\sum_{j=i+1}^{\eta} n_j}{i-1} - \binom{k-\sum_{j=i}^{\eta} n_j}{i-1}\right]
\end{aligned} \tag{C.1}$$

for $k > \eta \geq 3$. Since there is only one state in $L^0(\eta)$, $\mathbf{n} = (1, \ldots, 1)$, we define $\mathrm{pos}_\eta(\mathbf{n}, \eta) = 1$ for $\mathbf{n} \in L^0(\eta)$. Then, from Eq. (13) and Eq. (C.1) we have that the position in $\mathcal{A}^0$ of a state $\mathbf{n} \in L^0(k)$ is given by

$$\mathrm{pos}_{\mathcal{A}^0}(\mathbf{n}, \eta) = \mathrm{pos}_k(\mathbf{n}, \eta) + \sum_{\ell=\eta}^{k-1} L^0_\ell.$$

Appendix D

Approximation of the QSD with an auxiliary process

From any state $\mathbf{n} \in L^0(k)$ the process can only move to states in the adjacent levels $L^0(k-1)$ and $L^0(k+1)$, respectively. This means that the infinitesimal generator matrix of process $\mathcal{X}^{(j)}$, $\mathbf{Q}^{(j)}$ for $j = 1, 2$, is of quasi-birth and death type [7, 10], and can be written as

$$\mathbf{Q}^{(j)} = \begin{bmatrix} \mathbf{A}^{(j)}_{\eta,\eta} & \mathbf{A}^{(j)}_{\eta,\eta+1} & \mathbf{0} & \cdots & \mathbf{0} \\ \mathbf{A}^{(j)}_{\eta+1,\eta} & \mathbf{A}^{(j)}_{\eta+1,\eta+1} & \mathbf{A}^{(j)}_{\eta+1,\eta+2} & \cdots & \mathbf{0} \\ \mathbf{0} & \mathbf{A}^{(j)}_{\eta+2,\eta+1} & \mathbf{A}^{(j)}_{\eta+2,\eta+2} & \cdots & \mathbf{0} \\ \vdots & \vdots & \vdots & \ddots & \vdots \\ \mathbf{0} & \mathbf{0} & \mathbf{0} & \cdots & \mathbf{A}^{(j)}_{N-1,N} \\ \mathbf{0} & \mathbf{0} & \mathbf{0} & \cdots & \mathbf{A}^{(j)}_{N,N} \end{bmatrix}, \qquad \text{(D.1)}$$

where $\mathbf{0}$ are zero matrices of the appropriate sizes, and the $\mathbf{A}^{(j)}_{k,k'}$ sub-matrices are defined as follows: first, let $\mathbf{n}^i_k$ denote the state in $L^0(k)$ such that $\mathrm{pos}_k(\mathbf{n}^i_k, \eta) = i$, and $\mathbf{e}_\ell$ be the ℓ-th element of the standard basis. Then, we define the sub-matrices as

- For $\eta + 1 \le k \le N$

$$\left(\mathbf{A}^{(j)}_{k,k-1}\right)_{ip} = \begin{cases} \mu^{j,(\ell)}_{\mathbf{n}^i_k} & \text{if } \mathbf{n}^p_{k-1} = \mathbf{n}^i_k - \mathbf{e}_\ell \\ 0 & \text{otherwise.} \end{cases}$$

- for $\eta \le k \le N - 1$

$$\left(\mathbf{A}^{(j)}_{k,k+1}\right)_{ip} = \begin{cases} \lambda^{j,(\ell)}_{\mathbf{n}^i_k} & \text{if } \mathbf{n}^p_{k+1} = \mathbf{n}^i_k + \mathbf{e}_\ell, \\ 0 & \text{otherwise.} \end{cases}$$

- for $\eta \le k \le N$

$$\left(\mathbf{A}^{(j)}_{k,k}\right)_{ip} = \begin{cases} -\left(\sum_{\ell=1}^{\eta} \lambda^{j,(\ell)}_{\mathbf{n}^i_k} + \mu^{j,(\ell)}_{\mathbf{n}^i_k}\right) & \text{if } i = p, \\ 0 & \text{otherwise.} \end{cases}$$

Finally, given this quasi-birth and death matrix structure, the stationary probability distributions of $\mathcal{X}^{(1)}$ and $\mathcal{X}^{(2)}$ can be computed with a linear level-reduction algorithm [4], an outline of which is given in Algorithm D.1.

Algorithm D.1 Linear level-reduction algorithm to calculate the stationary probability distribution of the approximating process $\mathcal{X}^{(j)}$.

$\mathbf{H}_N^{(j)} = \mathbf{A}_{N,N}^{(j)}$
for $k = N-1, N-2, \ldots, \eta$ **do**
  $\mathbf{H}_k^{(j)} = \mathbf{A}_{k,k}^{(j)} - \mathbf{A}_{k,k+1}^{(j)} \left(\mathbf{H}_{k+1}^{(j)}\right)^{-1} \mathbf{A}_{k+1,k}^{(j)}$
end for
$\boldsymbol{\pi}_\eta^{(j)*} = 1$
for $k = \eta+1, \ldots, N$ **do**
  $\boldsymbol{\pi}_k^{(j)*} = -\boldsymbol{\pi}_{k-1}^{(j)*} \mathbf{A}_{k-1,k}^{(j)} \left(\mathbf{H}_k^{(j)}\right)^{-1}$
end for
for $k = \eta, \ldots, N$ **do**
  $\boldsymbol{\pi}_k^{(j)} = \frac{\boldsymbol{\pi}_k^{(j)*}}{\sum_{i=\eta}^{N} \sum_{j=1}^{L_i} \boldsymbol{\pi}_{i,j}^{(j)*}}$
end for

Appendix E

Certainty of absorption of $\mathcal{X}$

We show that the competition process reaches the absorbing state, $(0, \ldots, 0)$, with certainty using the method described in [9]. First, we partition the state space $\mathcal{S}$ into levels defined by

$$L(k) = \left\{ (n_1, \ldots, n_\eta) \in \mathcal{S} : \sum_{i=1}^{\eta} n_i = k \right\},$$

for $k = 0, 1, 2, \ldots$, so that $\mathcal{S} = \bigcup_{k=0}^{+\infty} L(k)$. Now, let us define

$$\lambda'_k = \max_{\mathbf{n} \in L(k)} \left\{ \sum_{i=1}^{\eta} \lambda_{\mathbf{n}}^{(i)} \right\}, \quad \mu'_k = \min_{\mathbf{n} \in L(k)} \left\{ \sum_{i=1}^{\eta} \mu_{\mathbf{n}}^{(i)} \right\}, \tag{E.1}$$

for $k \geq 1$, and since $L(0) = \{(0, \ldots, 0)\}$, we have that $\lambda'_0 = \mu'_0 = 0$. Equipped with these rates we can define a uni-variate birth and death process on the state space $\mathcal{S}' = \{L(k) : k = 0, 1, 2, \ldots\}$, which considers each level $L(k)$ as a single state, with birth rates λ'_k and death rates μ'_k (see Figure E.1). From the definition of these rates, this uni-variate birth and death process moves towards $L(0)$ at a slower rate than the original process [9]. Thus, if the birth and death process shown in Figure E.1 is absorbed at $L(0)$ with probability 1, then our competition process $\mathcal{X}$ is also absorbed with certainty.

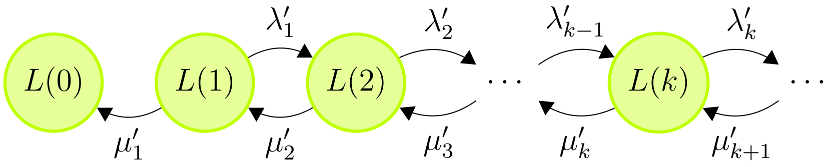

Figure E.1: Uni-variate competition process defined on the state space $\mathcal{S}' = \{L(k) : k = 0, 1, 2, \ldots\}$ with the rates defined in Eq. (E.1).

Theorem 3 of Reference [9] states that a sufficient condition for absorption at $L(0)$ to be certain is that the sum

$$\sum_{k=1}^{+\infty} \frac{1}{\lambda'_k \sigma_k}, \tag{E.2}$$

where $\sigma_k = \frac{\lambda'_1 \lambda'_2 \ldots \lambda'_{k-1}}{\mu'_2 \mu'_3 \ldots \mu'_k}$ for $k \geq 2$ and $\sigma_1 = 1$, is divergent.

First, notice that $\lambda_{\mathbf{n}}^{(i)}$ is bounded by

$$\begin{aligned}
\lambda_{\mathbf{n}}^{(i)} &= \varphi_i n_i \sum_{j=0}^{\eta-1} \sum_{k=1}^{\binom{\eta-1}{j}} p_{ij}^k e^{-\nu_{ij}^k} \sum_{r=0}^{M} \frac{\left(\nu_{ij}^k\right)^r}{r!} \frac{1}{n_i + \sum\limits_{l \in I_{ij}^k} n_l + r\langle n \rangle} \\
&\leq \varphi_i n_i \sum_{j=0}^{\eta-1} \sum_{k=1}^{\binom{\eta-1}{j}} p_{ij}^k e^{-\nu_{ij}^k} \sum_{r=0}^{+\infty} \frac{\left(\nu_{ij}^k\right)^r}{r!} \frac{1}{n_i} \\
&= \varphi_i n_i \sum_{j=0}^{\eta-1} \sum_{k=1}^{\binom{\eta-1}{j}} p_{ij}^k \frac{1}{n_i} = \varphi_i,
\end{aligned}$$

so that

$$\lambda'_k = \max_{\mathbf{n} \in L(k)} \left\{ \sum_{i=1}^{\eta} \lambda_{\mathbf{n}}^{(i)} \right\} \leq \sum_{i=1}^{\eta} \varphi_i, \tag{E.3}$$

and

$$\mu'_k = \min_{\mathbf{n} \in L(k)} \left\{ \sum_{i=1}^{\eta} \mu_{\mathbf{n}}^{(i)} \right\} = \min_{\mathbf{n} \in L(k)} \left\{ \sum_{i=1}^{\eta} \mu_i n_i \right\} = k\mu^*, \tag{E.4}$$

where $\mu^* = \min_{i=1,2,\ldots,\eta} \{\mu_i\}$. From Eq. (E.3) and Eq. (E.4) we obtain

$$\sum_{k=1}^{+\infty} \frac{1}{\lambda'_k \sigma_k} \geq \sum_{k=1}^{+\infty} \frac{k! \, (\mu^*)^{k-1}}{\left(\sum\limits_{i=1}^{\eta} \varphi_i \right)^k} = \sum_{k=1}^{+\infty} a_k, \tag{E.5}$$

and

$$\frac{a_{k+1}}{a_k} = \frac{(k+1)\mu^*}{\sum\limits_{i=1}^{\eta} \varphi_i} \to +\infty$$

as k increases. Thus by the ratio test this series diverges, which by Eq. (E.5) implies Eq. (E.2) also diverges, and by [9, Theorem 3] extinction of all clonotypes is certain at sufficiently late times.

Appendix F

Finite mean time to absorption of $\mathcal{X}$

Having shown that extinction is certain for the process in Appendix E, we can then analyse the time to extinction. Consider the state $\mathbf{n} = (n_1, \ldots, n_\eta) \in \mathcal{S}$ and let $\tau_{\mathbf{n}}$ be the mean time to extinction of all clonotypes, if the initial state is $\mathbf{n}$. Equivalently, $\tau_{\mathbf{n}}$ is the mean time to reach the absorbing state from state $\mathbf{n}$. Theorem 4 of Reference [9] states that $\tau_{\mathbf{n}}$ is finite for all $\mathbf{n}$ that have at least one non-zero entry if

$$\sum_{k=1}^{+\infty} \sigma_k < +\infty,$$

where the σ_i are the same as in Appendix E. Using Eq. (E.3) and Eq. (E.4) we obtain

$$\sum_{k=1}^{+\infty} \sigma_k \leq \sum_{k=1}^{+\infty} \frac{\left(\sum\limits_{i=1}^{\eta} \varphi_i\right)^k}{k!\,(\mu^*)^{k-1}} = \sum_{k=1}^{+\infty} b_k,$$

then

$$\frac{b_{k+1}}{b_k} = \frac{\sum\limits_{i=1}^{\eta} \varphi_i}{(k+1)\mu^*} \to 0,$$

as k increases and the series converges by the ratio test. Thus, by [9, Theorem 4] the mean time to extinction of all clonotypes from any non-zero initial state is finite. Furthermore, we can use the uni-variate process in Figure E.1 to find an upper bound on the mean time to extinction. Using the expression for the mean time to extinction of a uni-variate process [8], we can say that $\tau_{\mathbf{n}} \leq \tau'_m$ for $\mathbf{n} \in L(m)$, where

$$\tau'_m = \sum_{i=1}^{+\infty} \frac{1}{\lambda'_i \rho_i} + \sum_{j=1}^{m-1} \rho_j \sum_{k=j+1}^{+\infty} \frac{1}{\lambda'_k \rho_k},$$

with $\rho_1 = 1$, and $\rho_k = \prod_{i=0}^{k} \frac{\mu'_i}{\lambda'_i}$ for $k \geq 2$.

Appendix G

Mean time to first extinction event

In this section, we are interested in the mean time to the first extinction event. Let $\mathbf{X}(t)$ and W_j be random variables describing the state of the system at time t and the waiting time from the $j-1$-th event to the j-th event, respectively. We denote by $T_{\mathbf{n}}$ the random variable that describes the time to the first extinction event, starting from state $\mathbf{n} \in \mathcal{S} \setminus \mathcal{A}$. We have

$$T_{\mathbf{n}} = \inf \{ t \geq 0 : \; X_i(t) = 0 \text{ for some } i \mid \mathbf{X}(0) = \mathbf{n} \} .$$

Then, by the law of total probability we can express the mean time to extinction starting in state $\mathbf{n}$, $\mathbb{E}[T_{\mathbf{n}}]$, as

$$\hat{\tau}_{\mathbf{n}} \coloneqq \mathbb{E}[T_{\mathbf{n}}] = \mathbb{E}[W_1] + \sum_{\mathbf{m} \in \mathcal{S}} \mathbb{E}[T_{\mathbf{m}} | \mathbf{X}(0) = \mathbf{n}, \mathbf{X}(W_1) = \mathbf{m}] \, p_{\mathbf{nm}}, \tag{G.1}$$

where $p_{\mathbf{nm}}$ is the transition probability from $\mathbf{n}$ to $\mathbf{m}$ in the embedded Markov process [1]; that is, $p_{\mathbf{nm}}$ can be written as

$$p_{\mathbf{nm}} = \begin{cases} \frac{\lambda_{\mathbf{n}}^{(i)}}{\Delta_{\mathbf{n}}}, & \text{if } \mathbf{m} = \mathbf{n}^{(+i)}, \\ \frac{\mu_{\mathbf{n}}^{(i)}}{\Delta_{\mathbf{n}}}, & \text{if } \mathbf{m} = \mathbf{n}^{(-i)}, \\ 0, & \text{otherwise}, \end{cases} \tag{G.2}$$

where $\Delta_{\mathbf{n}} = \sum_{i=1}^{\eta} \left(\lambda_{\mathbf{n}}^{(i)} + \mu_{\mathbf{n}}^{(i)} \right)$. From Eq. (G.1) we can obtain the following set of difference equations

$$\hat{\tau}_{\mathbf{n}} = \frac{1}{\Delta_{\mathbf{n}}} + \sum_{i=1}^{\eta} \frac{\lambda_{\mathbf{n}}^{(i)}}{\Delta_{\mathbf{n}}} \hat{\tau}_{\mathbf{n}^{(+i)}} + \sum_{i=1}^{\eta} \frac{\mu_{\mathbf{n}}^{(i)}}{\Delta_{\mathbf{n}}} \hat{\tau}_{\mathbf{n}^{(-i)}} . \tag{G.3}$$

Thus, the mean time to extinction from state $\mathbf{n}$ is defined in terms of the mean time to extinction of its adjacent states. This set of equations also has the boundary condition that $\hat{\tau}_{\mathbf{n}} = 0$ if $n_i = 0$ for any $1 \leq i \leq \eta$. We can rewrite Eq. (G.3) as follows

$$-1 = -\Delta_{\mathbf{n}} \hat{\tau}_{\mathbf{n}} + \sum_{i=1}^{\eta} \lambda_{\mathbf{n}}^{(i)} \hat{\tau}_{\mathbf{n}^{(+i)}} + \sum_{i=1}^{\eta} \mu_{\mathbf{n}}^{(i)} \hat{\tau}_{\mathbf{n}^{(-i)}} .$$

It is easy to see that this system of equations can be represented as a matrix equation of the form $\mathbf{M} \boldsymbol{\tau} = \mathbf{1}$, from which we can solve for $\boldsymbol{\tau}$ numerically exploiting the fact that the matrix of coefficients $\mathbf{M}$ is sparse.

Appendix H

Calculation of the distribution of clonal sizes at the time of the first extinction event

To compute the probability of each clonotype to be the first one becoming extinct, first we truncate the infinite state space $\mathcal{S}$, following a similar approach to that in [5, 6]. In particular, we consider only states which have at most N_ε cells in total; that is, we consider a reflecting plane $\sum_{i=1}^{\eta} n_i = N_\varepsilon$, and define N_ε as the minimum value such that

$$\sum_{k=\eta}^{N_\varepsilon} \mathbb{P}\left(\mathbf{X}(t) \in L(k) : \ \forall t \geq 0\right) \geq 1 - \varepsilon, \tag{H.1}$$

is satisfied; that is, N_ε is the level of the QSD for which $\bigcup_{k=\eta}^{N_\varepsilon} L(k)$ captures at least $1-\varepsilon$ of the mass of the probability distribution. To compute N_ε we use a similar approach to that presented in [5, 6]. Since we will be calculating two different approximations of the QSD (see Section 3.1), we will choose N_ε to be the maximum between the values that satisfy Eq. (H.1) for each of the approximations.

Let us consider the subsets $\mathcal{A}^1 = \bigcup_{i=1}^{\eta} \mathcal{A}_i$ and $\mathcal{A}^0$, as defined in Eq. (B.5) and Eq. (12) respectively, and define the indicator function $\mathbf{1}_{\mathcal{A}^0}(\mathbf{n})$ as follows

$$\mathbf{1}_{\mathcal{A}^0}(\mathbf{n}) = \begin{cases} 1, & \text{if } \mathbf{n} \in \mathcal{A}^0, \\ 0, & \text{if } \mathbf{n} \in \mathcal{A}^1. \end{cases}$$

We then consider a modified competition process, $\breve{\mathcal{X}} = \left\{ \left(\breve{X}_1(t), \ldots, \breve{X}_\eta(t) \right) : t \geq 0 \right\}$, on the state space $\mathcal{A}^0 \cup \mathcal{A}^1$, with birth and death rates given by

$$\begin{aligned} \breve{\lambda}_{\mathbf{n}}^{(i)} &= \mathbf{1}_{\mathcal{A}^0}(\mathbf{n}) \cdot \lambda_{\mathbf{n}}^{(i)}, \\ \breve{\mu}_{\mathbf{n}}^{(i)} &= \mathbf{1}_{\mathcal{A}^0}(\mathbf{n}) \cdot \mu_{\mathbf{n}}^{(i)}; \end{aligned}$$

that is, this process behaves exactly as the original competition process $\mathcal{X}$ until a clonotype becomes extinct, at which point the process $\breve{\mathcal{X}}$ comes to an end. Let the random vector $\breve{\mathbf{X}}(t) = \left(\breve{X}_1(t), \ldots, \breve{X}_\eta(t) \right)$ describe the population of all clonotypes being modelled at

time t. We first define a position function for states in $\mathcal{A}_i$ (see Appendix I), which we use to define the general position function as follows

$$\mathrm{pos}_{\mathcal{A}^0\cup\mathcal{A}^1}(\mathbf{n},\eta) = \begin{cases} \mathrm{pos}_{\mathcal{A}^0}(\mathbf{n},\eta) & \text{if } \mathbf{n}\in\mathcal{A}^0, \\ \mathrm{pos}_{\mathcal{A}^1}(\mathbf{n},\eta) + \binom{N}{\eta} & \text{if } \mathbf{n}\in\mathcal{A}^1. \end{cases} \tag{H.2}$$

With the states ordered as indicated by Eq. (H.2), and if we define the embedded Markov process of $\breve{\mathcal{X}}$, $\mathcal{Y} = \{\mathbf{Y}(s) :\ s = 0, 1, 2, \ldots\}$, the transition matrix of $\mathcal{Y}$ is given by

$$\breve{\mathbf{P}} = \begin{bmatrix} \mathbf{P} & \mathbf{R} \\ \mathbf{0} & \mathbf{I} \end{bmatrix},$$

where $\mathbf{P}$ is a $|\mathcal{A}^0| \times |\mathcal{A}^0|$ matrix whose entries are the transition probabilities of states within $\mathcal{A}^0$, $\mathbf{R}$ is a $|\mathcal{A}^0| \times |\mathcal{A}^1|$ matrix whose entries are the transition probabilities from a state in $\mathcal{A}^0$ to a state in $\mathcal{A}^1$, $\mathbf{0}$ is the $|\mathcal{A}^1| \times |\mathcal{A}^0|$ zero matrix, and $\mathbf{I}$ is the identity matrix of size $|\mathcal{A}^1|$. Let $\mathbf{U}^{(s)}$ be the $|\mathcal{A}^0| \times |\mathcal{A}^1|$ matrix of probabilities to reach every absorbing state after at most s steps, where a step is defined as a birth or death event for any clonotype. That is, for a pair of states $\mathbf{n} \in \mathcal{A}^0$ and $\mathbf{m} \in \mathcal{A}^1$ we have

$$\mathbf{U}^{(s)}_{\mathrm{pos}_{\mathcal{A}^0}(\mathbf{n}),\mathrm{pos}_{\mathcal{A}^1}(\mathbf{m})} = \mathbb{P}\left\{\mathbf{Y}(s) = \mathbf{m} \mid \mathbf{Y}(0) = \mathbf{n}\right\}.$$

Then, we can write $\mathbf{U}^{(s)}$ as follows

$$\mathbf{U}^{(s)} = \left(\sum_{k=0}^{s-1} \mathbf{P}^k\right)\mathbf{R}.$$

If we make use of the fundamental matrix $\mathbf{W} = (\mathbf{I} - \mathbf{P})^{-1}$ associated to $\mathbf{P}$ [8], we find that

$$\mathbf{U} = \lim_{s\to+\infty} \mathbf{U}^{(s)} = \mathbf{W}\mathbf{R},$$

and thus, we can find the matrix of probabilities to reach each absorbing state in $\mathcal{A}^1$ given any initial state in $\mathcal{A}^0$, $\mathbf{U}$, by calculating

$$\mathbf{U} = (\mathbf{I} - \mathbf{P})^{-1}\mathbf{R}. \tag{H.3}$$

Note that the matrix $\mathbf{U}$ has a block structure and can be written as $\mathbf{U} = \begin{bmatrix}\mathbf{U}^1 & \mathbf{U}^2 & \cdots & \mathbf{U}^\eta\end{bmatrix}$, where $\mathbf{U}^i$ is the matrix of absorption probabilities from $\mathcal{A}^0$ to $\mathcal{A}_i$. That is, the entry in row ℓ and column j of $\mathbf{U}^i$, $(\mathbf{U}^i)_{\ell,j}$, is given by

$$\left(\mathbf{U}^i\right)_{\ell,j} = \lim_{s\to+\infty} \mathbb{P}\left\{\mathbf{Y}(s) = \mathbf{m} \mid \mathbf{Y}(0) = \mathbf{n}\right\},$$

where $\mathrm{pos}_{\mathcal{A}^0}(\mathbf{n}) = \ell$ and $\mathrm{pos}_{\mathcal{A}^1}(\mathbf{m}) = j$, for $1 \le \ell \le |\mathcal{A}^0|$ and $1 \le j \le |\mathcal{A}^1|$. Thus, each row of $\mathbf{U}$ adds up to 1 and represents the distribution of clonal sizes at the time of the first extinction event, with the initial state whose position is given by the row number. Furthermore, given the block structure of $\mathbf{U}$, each row of $\mathbf{U}^i$ contains the probabilities of clonal sizes for the surviving clonotypes when clonotype i is the first to become extinct.

To solve the matrix equation $(\mathbf{I}-\mathbf{P})\mathbf{U} = \mathbf{R}$, note that $\mathbf{I}-\mathbf{P}$ is of quasi-birth and death type and can be written as

$$\mathbf{I}-\mathbf{P} = \begin{bmatrix} \mathbf{I}_\eta & -\mathbf{B}_{\eta,\eta+1} & \cdots & \mathbf{0} \\ -\mathbf{B}_{\eta+1,\eta} & \mathbf{I}_{\eta+1} & \cdots & \mathbf{0} \\ \mathbf{0} & -\mathbf{B}_{\eta+2,\eta+1} & \cdots & \mathbf{0} \\ \vdots & \vdots & \ddots & \vdots \\ \mathbf{0} & \mathbf{0} & \cdots & -\mathbf{B}_{N_\varepsilon-1,N_\varepsilon} \\ \mathbf{0} & \mathbf{0} & \cdots & \mathbf{I}_{N_\varepsilon} \end{bmatrix}, \tag{H.4}$$

where $\mathbf{I}_k$ is the identity matrix of order $L_k^0 \times L_k^0$, $\mathbf{0}$ are zero matrices of the appropriate sizes, and the $\mathbf{B}_{k,k'}$ matrices are defined as follows: let $\mathbf{n}_k^i$ denote the state in $L^0(k)$ such that $\text{pos}_k(\mathbf{n}_k^i, \eta) = i$, then

- for $\eta+1 \leq k \leq N_\varepsilon$
$$(\mathbf{B}_{k,k-1})_{ij} = p_{\mathbf{n}_k^i \mathbf{n}_{k-1}^j},$$
- for $\eta \leq k \leq N_\varepsilon - 1$
$$(\mathbf{B}_{k,k+1})_{ij} = p_{\mathbf{n}_k^i \mathbf{n}_{k+1}^i},$$

where $p_{\mathbf{n}_k^i \mathbf{n}_{k-1}^j}$ and $p_{\mathbf{n}_k^i \mathbf{n}_{k+1}^i}$ are the transition probabilities given by Eq. (G.2). The matrix $\mathbf{R}$ has the form $\mathbf{R} = \begin{bmatrix}\mathbf{R}^1 & \mathbf{R}^2 & \cdots & \mathbf{R}^\eta\end{bmatrix}$, where $\mathbf{R}^i$ is the matrix of transition probabilities from $\mathcal{A}^0$ to $\mathcal{A}_i$. The $\mathbf{R}^i$ matrices are diagonal by blocks and can be written as

$$\mathbf{R}^i = \begin{bmatrix} \mathbf{R}^i_{\eta,\eta-1} & \mathbf{0} & \mathbf{0} & \cdots & \mathbf{0} \\ \mathbf{0} & \mathbf{R}^i_{\eta+1,\eta} & \mathbf{0} & \cdots & \mathbf{0} \\ \mathbf{0} & \mathbf{0} & \mathbf{R}^i_{\eta+2,\eta+1} & \cdots & \mathbf{0} \\ \vdots & \vdots & \vdots & \ddots & \vdots \\ \mathbf{0} & \mathbf{0} & \mathbf{0} & \cdots & \mathbf{R}^i_{N_\varepsilon,N_\varepsilon-1} \end{bmatrix}.$$

We now define the $\mathbf{R}^i_{k,k-1}$ matrices. Let us first introduce $\mathbf{n}_k^\ell$ to denote the state in $L^0(k)$ such that $\text{pos}_k(\mathbf{n}_k^\ell, \eta) = \ell$, and $\mathbf{m}_{i,k}^\ell$ denote the state in $L^1(k) \subset \mathcal{A}_i$, such that $\text{pos}_k(\hat{\mathbf{m}}_{i,k}^\ell, \eta-1) = \ell$. Then, for $\eta \leq k \leq N_\varepsilon$, we define

$$(\mathbf{R}^i_{k,k-1})_{ab} = p_{\mathbf{n}_k^a \mathbf{m}_{i,k-1}^b},$$

where $p_{\mathbf{n}_k^a \mathbf{m}_{i,k-1}^b}$ is the transition probability given by Eq. (G.2). The structure of $\mathbf{U}$ allows us to make use of a linear level-reduction algorithm to solve Eq. (H.3) one $\mathbf{U}^i$ block at a time. An outline of the algorithm is given in Algorithm H.1. Finally, making use of the structure of $\mathbf{U}$, the probability of clonotype i being the first to become extinct starting in state $\mathbf{n}_0 \in \mathcal{A}^0$, denoted by $\mathcal{U}^i_{\mathbf{n}_0}$, can be calculated as follows

$$\mathcal{U}^i_{\mathbf{n}_0} = \sum_{j=1}^{|\mathcal{A}_i|} \left(\mathbf{U}^i\right)_{\text{pos}_{\mathcal{A}^0}(\mathbf{n}_0),j}, \tag{H.5}$$

where $\left(\mathbf{U}^i\right)_{\ell,j}$ denotes the entry of the matrix $\mathbf{U}^i$ in the ℓ-th row and j-th column.

Algorithm H.1 Linear level-reduction algorithm to calculate $\mathbf{U}$.

```
H_{N_ε} = I_{L^0_{N_ε}}
for k = N_ε − 1, N_ε − 2, ..., η do
    H_k = I_{L^0_k} − B_{k,k+1} H^{-1}_{k+1} B_{k+1,k}
end for
for i = 1, 2, ..., η do
    for j = η − 1, η, ..., N_ε − 1 do
        K^i_{N_ε,j} = R^i_{N_ε,j}
        for k = N_ε − 1, N_ε − 2, ..., η do
            K^i_{k,j} = B_{k,k+1} H^{-1}_{k+1} K^i_{k+1,j} + R^i_{k,j}
        end for
        U^i_{η,j} = H^{-1}_η K^i_{η,j}
        for k = η + 1, η + 2, ..., N_ε do
            U^i_{k,j} = H^{-1}_k (K^i_{k,j} + B_{k,k-1} U^i_{k-1,j})
        end for
    end for
end for
```

Appendix I

Position of states within levels with a single extinct clonotype

We start by ordering the absorbing states as follows

$$\mathcal{A}_1 \prec \mathcal{A}_2 \prec \cdots \prec \mathcal{A}_{\eta-1} \prec \mathcal{A}_\eta, \tag{I.1}$$

and noting that we can bijectively project these sets onto a space of dimension $\eta - 1$ by removing the i-th element (which is the population of the clonotype that has become extinct). We will denote the projected element by $\hat{\mathbf{n}}$ and the projected set by $\hat{\mathcal{A}}_i$. Given that $\hat{\mathcal{A}}_i$ has the same structure as $\mathcal{A}^0$, we can organise it in ordered levels as follows

$$L^1(\eta-1) \prec L^1(\eta) \prec L^1(\eta+1) \prec \cdots \prec L^1(N_\varepsilon - 1).$$

The last level we can consider is $N_\varepsilon - 1$, since for a state to reach $L^1(N_\varepsilon)$ there must have been an extinction event from a state in $L^0(N_\varepsilon + 1)$, which is not part of the state space for the process with the reflecting boundary. The number of states in level k is given by

$$L^1_k = |L^1(k)| = \binom{k-1}{\eta-2},$$

and the total number of states in $\hat{\mathcal{A}}_i$ under the reflecting boundary is

$$|\hat{\mathcal{A}}_i| = \sum_{\ell=\eta-1}^{N_\varepsilon-1} L^1_\ell = \sum_{\ell=\eta-1}^{N_\varepsilon-1} \binom{\ell-1}{\eta-2} = \binom{N_\varepsilon-1}{\eta-1}. \tag{I.2}$$

Then, the position in $\hat{\mathcal{A}}_i$ of a state $\hat{\mathbf{n}} \in L^1(k)$ is given by

$$\mathrm{pos}_{\hat{\mathcal{A}}_i}(\hat{\mathbf{n}}, \eta-1) = \mathrm{pos}_k(\hat{\mathbf{n}}, \eta-1) + \sum_{i=\eta-1}^{k-1} L^1_i,$$

and by reversing the projection of $\mathcal{A}_i$ onto $\hat{\mathcal{A}}_i$ we have

$$\mathrm{pos}_{\mathcal{A}_i}(\mathbf{n}, \eta) = \mathrm{pos}_{\hat{\mathcal{A}}_i}(\hat{\mathbf{n}}, \eta-1). \tag{I.3}$$

From Eq. (I.1), Eq. (I.2), and Eq. (I.3) we have that the position of state $\mathbf{n} \in L^1(k) \subset \mathcal{A}_i$ in $\mathcal{A}^1$ is given by

$$\mathrm{pos}_{\mathcal{A}^1}(\mathbf{n}, \eta) = \mathrm{pos}_{\mathcal{A}_i}(\mathbf{n}, \eta) + (i-1)|\hat{\mathcal{A}}_i|$$

Appendix J

Calculation of the distribution of divisions before extinction of a clonotype

The distribution of divisions before extinction is an indicator of how much proliferation takes place before the extinction of a T cell clonal family. To calculate this distribution probability we use the truncated state space defined in Appendix H. Given a fiducial (fixed but arbitrary) initial state, $\mathbf{n} \in \mathcal{A}^0$, we denote by $\mathcal{D}_{i,d}^{(s)}(\mathbf{n})$ the probability that clonotype i divided d times in at most s steps before becoming extinct if it had an initial size of $\mathbf{n}$. We write

$$\mathcal{D}_{i,d}^{(s)}(\mathbf{n}) = \mathbb{P}\left(\begin{matrix}\text{clone } i \text{ divided } d \text{ times in at most} \\ s \text{ steps before becoming extinct}\end{matrix} \middle| \mathbf{X}(0) = \mathbf{n}\right). \tag{J.1}$$

Since a cell of clonotype i can divide after the extinction of other clonotypes, we must consider the complete state space, $\mathcal{S}$, with a reflecting boundary at $\sum_{i=1}^{\eta} n_i = N_\varepsilon$. To order the elements of $\mathcal{S}$, note that we can bijectively map an element $\mathbf{n} \in \mathcal{S}$ to $\mathcal{A}^0$ by adding $\mathbf{e} = (1, \dots, 1)$ to $\mathbf{n}$. Then, the position of state $\mathbf{n}$ in the k-th level of $\mathcal{S}$, is given by

$$\mathrm{pos}_{\mathcal{S},k}(\mathbf{n}, \eta) = \mathrm{pos}_{k+\eta}(\mathbf{n} + \mathbf{e}, \eta).$$

With this bijection we can make use of the levels defined in $\mathcal{A}^0$ (see Eq. (C.1)). We consider $\mathcal{D}_{i,d}^{(s)}$ as the vector built from entries $\mathcal{D}_{i,d}^{(s)}(\mathbf{n})$, for each $\mathbf{n} \in \mathcal{S}$. We make use of the level structure to divide it into sub-vectors corresponding to the states in each level of $\mathcal{S}$, that is

$$\mathcal{D}_{i,d}^{(s)} = \left[\mathcal{D}_{i,d,0}^{(s)}, \mathcal{D}_{i,d,1}^{(s)}, \dots, \mathcal{D}_{i,d,N_\varepsilon}^{(s)}\right]^\top,$$

where $\mathcal{D}_{i,d,k}^{(s)}$ is the vector of probabilities for $\mathbf{n} \in L(k)$. Since we are interested in the probability of clone i dividing, we will not consider the usual transition matrix, but instead we will separate it into the vector of probabilities for division in clonotype i, and the sub-stochastic matrix $\mathbf{P}^{(i)}$ of all transition probabilities except those for division in

clonotype i; that is,

$$\mathbf{P}^{(i)} = \begin{bmatrix} \mathbf{0} & \mathbf{0} & \mathbf{0} & \mathbf{0} & \cdots & \mathbf{0} \\ \mathbf{C}^{(i)}_{1,0} & \mathbf{0} & \mathbf{C}^{(i)}_{1,2} & \mathbf{0} & \cdots & \mathbf{0} \\ \mathbf{0} & \mathbf{C}^{(i)}_{2,1} & \mathbf{0} & \mathbf{C}^{(i)}_{2,3} & \cdots & \mathbf{0} \\ \vdots & \vdots & \vdots & \vdots & \ddots & \vdots \\ \mathbf{0} & \mathbf{0} & \mathbf{0} & \mathbf{0} & \cdots & \mathbf{C}^{(i)}_{N_\varepsilon-1,N_\varepsilon} \\ \mathbf{0} & \mathbf{0} & \mathbf{0} & \mathbf{0} & \cdots & \mathbf{0} \end{bmatrix}, \tag{J.2}$$

where $\mathbf{0}$ are zero matrices of the appropriate sizes and the $\mathbf{C}^{(i)}_{k,k'}$ matrices are defined as follows: let $\mathbf{n}^\ell_k$ denote the state in $L(k)$ such that $\mathrm{pos}_{\mathcal{S},k}(\mathbf{n}^\ell_k, \eta) = \ell$, then

- for $1 \le k \le N_\varepsilon$

$$\left(\mathbf{C}^{(i)}_{k,k-1}\right)_{\ell j} = \begin{cases} p_{\mathbf{n}^\ell_k \mathbf{n}^j_{k-1}} & \text{if } \left(\mathbf{n}^\ell_k\right)_i > 0, \\ 0 & \text{otherwise.} \end{cases}$$

- for $1 \le k \le N_\varepsilon - 1$

$$\left(\mathbf{C}^{(i)}_{k,k+1}\right)_{\ell j} = \begin{cases} p_{\mathbf{n}^\ell_k \mathbf{n}^j_{k+1}} & \text{if } \left(\mathbf{n}^\ell_k\right)_i > 0 \text{ and } \left(\mathbf{n}^\ell_k\right)_i = \left(\mathbf{n}^j_{k+1}\right)_i, \\ 0 & \text{otherwise.} \end{cases}$$

We note that given that we are calculating the probability of division before extinction, all transition probabilities for a state in which clonotype i is already extinct are zero. We calculate the distribution recursively on the number of divisions before extinction. Thus, we first define the vector $\mathbf{d}^{(i)}_0$ as the vector containing the probability that in zero steps clonotype i will become extinct. We write

$$\left(\mathbf{d}^{(i)}_0\right)_{\mathrm{pos}_{\mathcal{S},k}(\mathbf{n},\eta)} = \begin{cases} 1, & \text{if } n_i = 0, \\ 0, & \text{otherwise.} \end{cases}$$

Then, the probability that clone i will not divide before becoming extinct in s or fewer steps is given by

$$\mathcal{D}^{(s)}_{i,0} = \left(\sum_{j=0}^{s} \left(\mathbf{P}^{(i)}\right)^j \right) \mathbf{d}^{(i)}_0.$$

If we make use of the fundamental matrix associated to $\mathbf{P}^{(i)}$, $\mathbf{W}^{(i)} = \left(\mathbf{I} - \mathbf{P}^{(i)}\right)^{-1}$, we have

$$\mathcal{D}_{i,0} = \lim_{t\to+\infty} \mathcal{D}^{(s)}_{i,0} = \mathbf{W}^{(i)} \mathbf{d}^{(i)}_0,$$

which leads to

$$\left(\mathbf{I} - \mathbf{P}^{(i)}\right) \mathcal{D}_{i,0} = \mathbf{d}^{(i)}_0.$$

This expression allows us to solve for the probability of extinction of clonotype i after 0 divisions, $\mathcal{D}_{i,0}$:

$$\mathcal{D}_{i,0} = \left(\mathbf{I} - \mathbf{P}^{(i)}\right)^{-1} \mathbf{d}^{(i)}_0. \tag{J.3}$$

For $\ell > 0$ divisions, we define the vector containing the probability of clonotype i becoming extinct after ℓ divisions given that it has divided once, as follows

$$\left(\mathbf{d}_{\ell}^{(i)}\right)_{\mathrm{pos}_{\mathcal{S},k}(\mathbf{n},\eta)} = p_{\mathbf{n}\mathbf{n}^{(+i)}} \mathcal{D}_{i,\ell-1}\left(\mathbf{n}^{(+i)}\right).$$

Then, the vector of probabilities of clonotype i becoming extinct after ℓ divisions, $\mathcal{D}_{i,\ell}$, satisfies the recursive equation

$$\mathcal{D}_{i,\ell} = \left(\mathbf{I} - \mathbf{P}^{(i)}\right)^{-1} \mathbf{d}_{\ell}^{(i)}. \tag{J.4}$$

Finally, with the use of an adapted version of Algorithm H.1, we can solve Eq. (J.3) and Eq. (J.4) recursively until the number of divisions reaches $\ell_{\mathbf{n},\delta}$, such that

$$\sum_{d=0}^{\ell_{\mathbf{n},\delta}} \mathcal{D}_{i,d}(\mathbf{n}) \geq 1 - \delta,$$

with $\mathbf{n}$ the initial state under consideration. The values $\mathcal{D}_{i,d}(\mathbf{n})$ for $d = 1, 2, \ldots, \ell_{\mathbf{n},\delta}$ describe the probability distribution for the number of divisions of clonotype i before its extinction with initial state $\mathbf{n}$.

Bibliography

[1] Linda JS Allen. *An introduction to stochastic processes with applications to biology.* CRC Press, Lubbock, TX, 2 edition, 2010.

[2] Lee J Bain and Max Engelhardt. *Introduction to probability and mathematical statistics.* Duxbury Press, Belmont, CA, 2000.

[3] John N Darroch and Eugene Seneta. On quasi-stationary distributions in absorbing continuous-time finite markov chains. *Journal of Applied Probability*, 4(1):192–196, 1967.

[4] DP Gaver, PA Jacobs, and Guy Latouche. Finite birth-and-death models in randomly changing environments. *Advances in applied probability*, 16(4):715–731, 1984.

[5] Antonio Gómez-Corral and M López García. On the number of births and deaths during an extinction cycle, and the survival of a certain individual in a competition process. *Computers & Mathematics with Applications*, 64(3):236–259, 2012.

[6] Antonio Gómez-Corral and M López García. Extinction times and size of the surviving species in a two-species competition process. *Journal of mathematical biology*, 64(1):255–289, 2012.

[7] Antonio Gómez-Corral and M López-García. Perturbation analysis in finite LD-QBD processes and applications to epidemic models. *Numerical linear algebra with applications*, 25(5):e2160, 2018.

[8] Taylor Howard M and Samuel Karlin. *An introduction to stochastic modeling.* Academic Press, Oxford, 3 edition, 1998.

[9] Donald L Iglehart. Multivariate competition processes. *The Annals of Mathematical Statistics*, 35:350–361, 1964.

[10] Vidyadhar G Kulkarni. *Modeling and analysis of stochastic systems.* CRC Press, New York, 3 edition, 2017.

[11] Emily R Stirk, Carmen Molina-París, and Hugo A van den Berg. Stochastic niche structure and diversity maintenance in the T cell repertoire. *Journal of theoretical biology*, 255:237–249, 2008.
